# Design, Synthesis, and Biological Evaluation of Pyridazinones Containing the (2-Fluorophenyl) Piperazine Moiety as Selective MAO-B Inhibitors

**DOI:** 10.3390/molecules25225371

**Published:** 2020-11-17

**Authors:** Muhammed Çeçen, Jong Min Oh, Zeynep Özdemir, Saliha Ebru Büyüktuncel, Mehtap Uysal, Mohamed A. Abdelgawad, Arafa Musa, Nicola Gambacorta, Orazio Nicolotti, Bijo Mathew, Hoon Kim

**Affiliations:** 1 Department of Pharmaceutical Chemistry, Faculty of Pharmacy, Inonu University, Malatya 44280, Turkey; mcecen2344@gmail.com; 2 Department of Pharmacy, and Research Institute of Life Pharmaceutical Sciences, Sunchon National University, Suncheon 57922, Korea; ddazzo005@naver.com; 3 Department of Analytical Chemistry, Faculty of Pharmacy, Inonu University, Malatya 44280, Turkey; saliha.buyuktuncel@inonu.edu.tr; 4 Department of Pharmaceutical Chemistry, Faculty of Pharmacy, Gazi University, Ankara 06100, Turkey; mgokce99@gmail.com; 5 Department of Pharmaceutical Chemistry, College of Pharmacy, Jouf University, Sakaka 72341, Al Jouf, Saudi Arabia; mohamedabdelwahab976@yahoo.com; 6 Department of Pharmaceutical Organic Chemistry, Faculty of Pharmacy, Beni-Suef University, Beni Suef 62514, Egypt; 7 Department of Pharmacognosy, College of Pharmacy, Jouf University, Sakaka 72341, Al Jouf, Saudi Arabia; akmusa@ju.edu.sa; 8 Department of Pharmacognosy, Faculty of Pharmacy, Al-Azhar University, Cairo 11371, Egypt; 9 Dipartimento di Farmacia-Scienze del Farmaco, Università degli Studi di Bari “Aldo Moro”, Via E. Orabona, 4, I-70125 Bari, Italy; nicola.gambacorta1@uniba.it (N.G.); orazio.nicolotti@uniba.it (O.N.); 10 Department of Pharmaceutical Chemistry, Amrita School of Pharmacy, Amrita Vishwa Vidyapeetham, Amrita Health Science Campus, Kochi 682 041, India

**Keywords:** monoamine oxidase, pyridazinone, kinetics, reversibility, ADME, molecular docking

## Abstract

Twelve pyridazinones (**T1**–**T12**) containing the (2-fluorophenyl) piperazine moiety were designed, synthesized, and evaluated for monoamine oxidase (MAO) -A and -B inhibitory activities. **T6** was found to be the most potent MAO-B inhibitor with an IC_50_ value of 0.013 µM, followed by **T3** (IC_50_ = 0.039 µM). Inhibitory potency for MAO-B was more enhanced by *meta* bromo substitution (**T6**) than by *para* bromo substitution (**T7**). For *para* substitution, inhibitory potencies for MAO-B were as follows: -Cl (**T3**) > -N(CH_3_)_2_ (**T12**) > -OCH_3_ (**T9**) > Br (**T7**) > F (**T5**) > -CH_3_ (**T11**) > -H (**T1**). **T6** and **T3** efficiently inhibited MAO-A with IC_50_ values of 1.57 and 4.19 µM and had the highest selectivity indices (SIs) for MAO-B (120.8 and 107.4, respectively). **T3** and **T6** were found to be reversible and competitive inhibitors of MAO-B with K_i_ values of 0.014 and 0.0071, respectively. Moreover, **T6** was less toxic to healthy fibroblast cells (L929) than **T3**. Molecular docking simulations with MAO binding sites returned higher docking scores for **T6** and **T3** with MAO-B than with MAO-A. These results suggest that **T3** and **T6** are selective, reversible, and competitive inhibitors of MAO-B and should be considered lead candidates for the treatment of neurodegenerative disorders like Alzheimer’s disease.

## 1. Introduction

Alzheimer’s disease (AD) is a fatal neurodegenerative disease characterized by progressive cognitive dysfunctions, an inability to perform daily activities, and by the various neuropsychiatric symptoms of late-stage AD [1]. AD is also associated with a reduction in the number of neurons in the limbic system, which manages memory processes [2]. Furthermore, the levels of many neurotransmitters, especially acetylcholine, are diminished in cerebral cortex in concert with neuronal loss, and the levels of other neurotransmitters, such as cholinergic, noncholinergic, serotonergic, dopaminergic, and neuropeptidergic neurotransmitters are altered in AD [3].

It has been reported that monoamine oxidase-B (MAO-B) activity is enhanced in AD, particularly in reactive astrocytes near β-amyloid (Aβ) plaques [4]. In a more recent study, it was reported that the MAO-B levels were elevated in astrocytes and in pyramidal neurons in the frontal cortex, hippocampus CA1 region, and entorhinal cortex [5]. MAO-B inhibitors can reduce neuronal damage by inhibiting oxidative deamination, and it is believed that MAO-B inhibition reduces oxidative stress and neurodegeneration, and thereby, delays AD progression [6,7,8]. Many scaffolds, such as chalcones, chromones, coumarins, thiazolidinones, isatins, and pyrazolines, have been reported to inhibit MAO-B selectively and to exhibit promising neuroprotective properties [9,10,11,12,13,14,15,16,17,18,19,20,21,22]. In addition, MAO-B inhibitors have been recently designed with two aryl or heteroaryl hydrophobic rings separated by short enamide, unsaturated ketone, multi-conjugated ketone, amides, or hydrazone spacers [23,24,25,26,27].

Pyridazinones are pyridazine-containing heterocycles with two adjacent ring nitrogen atoms and keto functionality. Several FDA-approved drugs with a pyridazinone nucleus, such as pimobendan, bemoradan, indolidan, and levosimendan, have been commercialized over the past 30 years as antihypertensive, and azanrinone and emorfazone are now marketed as cardiotonic and anti-inflammatory agents, respectively [28]. Recently, our group explored a novel class of pyridazinone hydrazides with pendant piperazine moieties and investigated their abilities to competitively and reversibly inhibit MAO-B [29].

Here, we report the MAO-A and MAO-B inhibitory effects of a series of novel hydrazone derivatives (**T1**–**T12**) bearing a phenyl ring with different substituents. We previously reported the anticancer activities of seven of these derivatives (**T1**–**T7**) [30]. The other five (**T8**–**T12**) were synthesized during the present study. Lead compounds were further examined by investigating enzyme inhibition kinetics, reversibility and cytotoxicity to normal cell lines. In addition, binding modes to MAO-A and MAO-B and interactions with their active sites were determined by molecular docking simulations. Structure-activity relationships were investigated by comparing in vitro enzyme inhibition and molecular docking results.

## 2. Results and Discussion

### 2.1. Chemistry

**Scheme 1:**
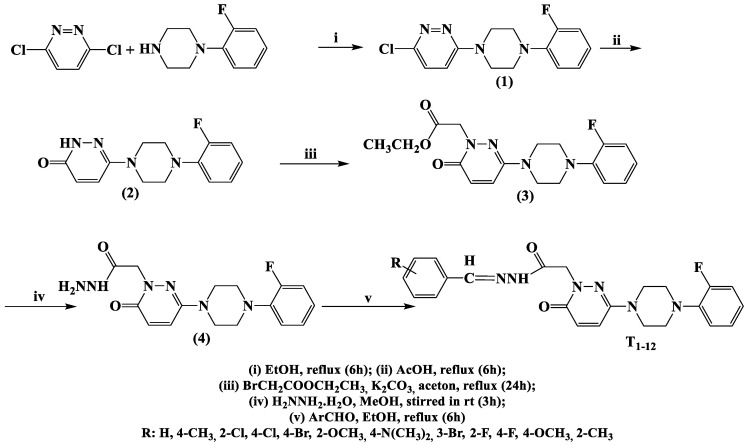
Synthetic pathway for the production of 6-(4-(2-fluorophenyl) piperazine-1-yl)-3(*2H*)-pyridazinone-2-acetyl-2-(substituted/non-substituted benzal)hydrazone derivatives (**T1**–**T12**).

The synthetic routes used to produce the 12 derivatives are shown in Scheme 1. Compounds were synthesized by reacting commercial 3,6-dichloropyridazine with (2-fluorophenyl)piperazine in ethanol to afford 3-chloro-6-[4-(2-fluorophenyl)piperazine-1-yl]pyridazine. The physical and spectral properties of 3-chloro-6-[4-(2-fluorophenyl)piperazine-1-yl]pyridazine concurred with literature data [30]. Compound 6-(4-(2-fluorophenyl)piperazine-1-yl)-3(*2H*)-pyridazinone was obtained by hydrolyzing 3-chloro-6-substituted pyridazine in hot glacial acetic acid. Ethyl 6-(4-(2-fluorophenyl)piperazine-1-yl)-3(*2H*)-pyridazinone-2-yl-acetate was obtained by reacting 6-(4-(2-fluorophenyl)piperazine-1-yl)-3(*2H*)-pyridazinone with ethyl bromoacetate in acetone in the presence of K_2_CO_3_. Then, 6-(4-(2-fluorophenyl)piperazine-1-yl)-3(*2H*)-pyridazinone-2-yl-acetohydrazide was synthesized by condensing ethyl 6-(4-(2-fluorophenyl)piperazine-1-yl)-3(*2H*)-pyridazinone-2-yl-acetate with hydrazine hydrate (yield 99%). The title compounds **T1**–**T7**, which contain benzalhydrazone, were obtained by condensing 6-(4-(2-fluorophenyl)piperazine-1-yl)-3(*2H*)-pyridazinone-2-yl-acetohydrazide with substituted/non-substituted benzaldehydes, as we previously described [31]. The other five compounds (**T8**–**12**) were synthesized using the same methods and are reported for the first time. The molecular structures of these compounds were confirmed by ^1^H-NMR, ^13^C-NMR, and HRMS (Supplementary Materials). The molecular structures, yields, melting points, molecular weights, and molecular formulas of **T1**–**T12** are provided in Table 1.

**Figure 1:**
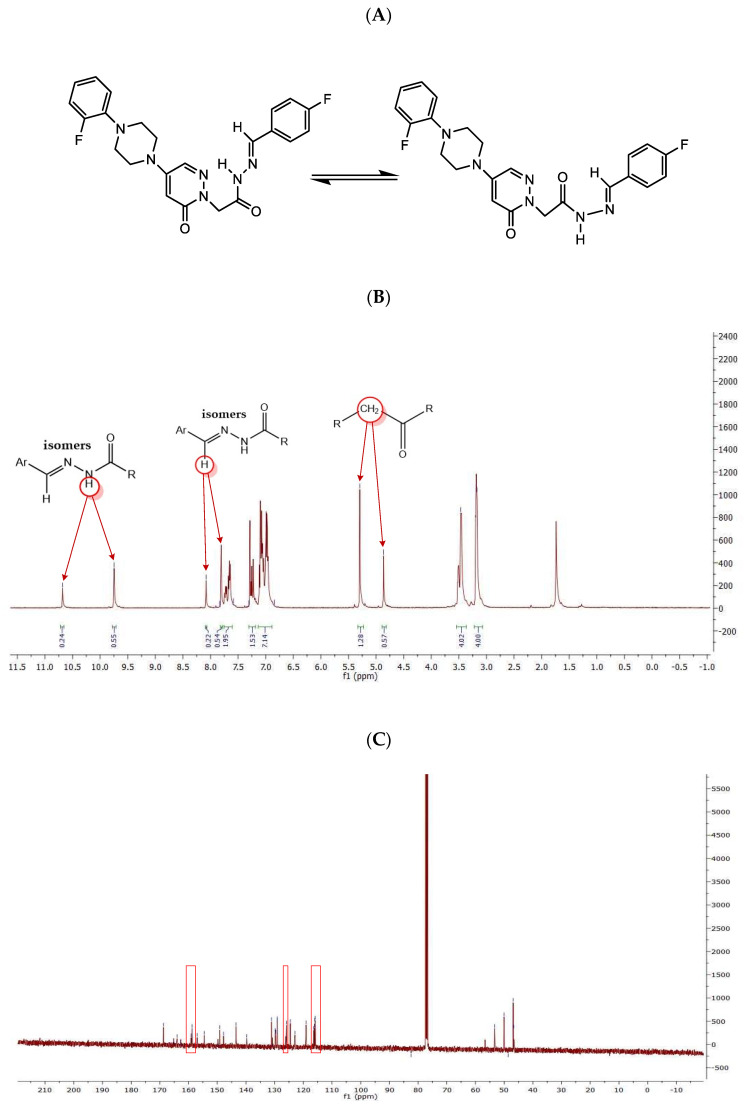
Structures of two conformational isomers (**A**), and ^1^H-NMR (**B**) and ^13^C-NMR (**C**) spectra of **T10**.

It was observed that these compounds were in two stereoisomeric forms by interpretation of NMR spectra. For example, **T10** existed as two stereoisomeric forms in ^1^H-NMR spectrum (Figure 1A), indicating the resonance of methylene attached to the carbonyl group at 5.30 and 4.87 ppm (2H; s; CH_2_CO) and two pairs of singlet (one pair at 8.0 and 8.4 ppm and another one at 9.5 and 10.6 ppm) (Figure 1B). In addition, the carbons of pyridazinone C^6^, pyridazinone C^4^, and 2-fluorophenyl C^5^ were double in ^13^C-NMR spectrum at low MHz (Figure 1C). These pairs of signals clearly show that these compounds exist in two stereoisomeric forms, as other isomers described previously [32,33].

### 2.2. MAO Inhibitory Activities

Of the 12 derivatives, six potently inhibited MAO-B with residual activities of <50% at 1.0 µM (Table 2). **T6** most potently inhibited MAO-B, with an IC_50_ value of 0.013 µM, and was followed by **T3** and **T4** (IC_50_ = 0.039 and 0.099 µM, respectively). **T2**, **T12**, and **T9** also potently inhibited MAO-B (IC_50_ = 0.10, 0.15, and 0.20 µM, respectively). Bromine at the *meta* position (**T6**) increased MAO-B inhibitory activity more so than at the *para* position (**T7**). Regarding substituents at the *para* position, MAO-B inhibitory activities increased in the order: –Cl (**T3**) > –N(CH_3_)_2_ (**T12**) > -OCH_3_ (**T9**) > -Br (**T7**) > -F (**T5**) > -CH_3_ (**T11**) > -H (**T1**). In addition, **T6** efficiently inhibited MAO-A with an IC_50_ value of 1.57 µM, followed by **T3** (IC_50_ = 4.19 µM) (Table 2), and **T6** also had the highest selectivity index (SI) value (120.8) for MAO-B, followed by **T3** (SI = 107.4).

The presence of different substituents on the phenyl hydrazone ring did not appreciably impact MAO selectivity, whereas the introduction of halogens, methoxy, methyl, or dimethylamino groups reduced MAO-B inhibition versus non-substituted hydrazone. The lead compounds **T6** and **T3**, which had methoxy or chlorine groups at the *ortho* position of the phenyl system, were 11 and 3 times more potent, respectively, than the reference reversible MAO-B inhibitor lazabemide. Furthermore, **T6** was twice as potent as the reference irreversible MAO-B inhibitor pargyline. Shifting methoxy group from the *ortho* to the *para* position of the phenyl ring (**T11**) dramatically reduced MAO-B inhibition (IC_50_ = 6.86 ± 0.37 µM) and shifting chlorine from the *ortho* to the *para* (**T4**) reduced MAO-B inhibition. These observations indicate that *ortho* is preferred over *para* in terms of MAO-B inhibition. Interestingly, recent studies have reported that chlorine and methoxy groups have considerable impacts on MAO-B inhibition [34,35].

### 2.3. Kinetics of MAO-B Inhibitions

**Figure 2:**
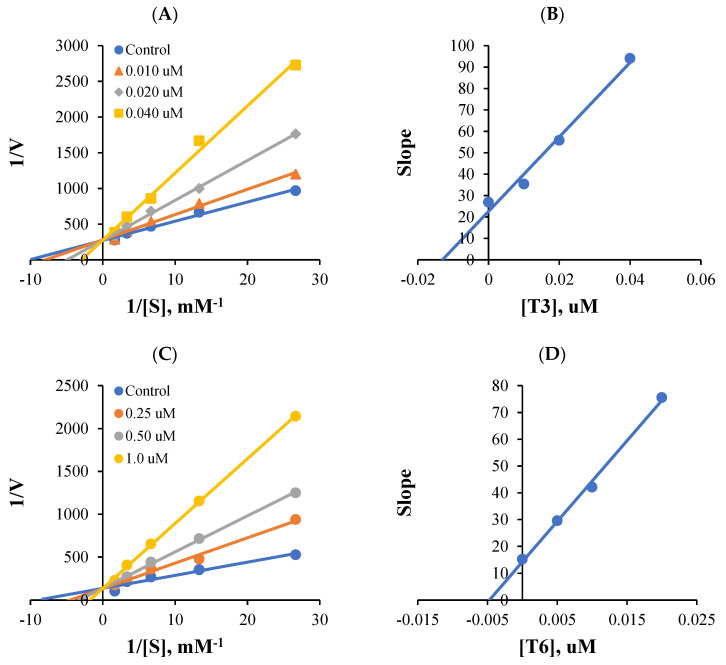
Lineweaver-Burk plots for MAO-B inhibition by **T3** or **T6** (**A**,**C**), and respective secondary plots (**B**,**D**) of slopes vs. inhibitor concentrations.

Lineweaver-Burk plots and secondary plots obtained for MAO-B inhibition showed that **T3** and **T6** were competitive inhibitors (Figure 2A,C), with K_i_ values of 0.014 ± 0.0014 and 0.0071 ± 0.0033 µM, respectively (Figure 2B,D). These results suggest that **T3** and **T6** are competitive inhibitors of MAO-B.

### 2.4. Reversibility Studies

**Figure 3:**
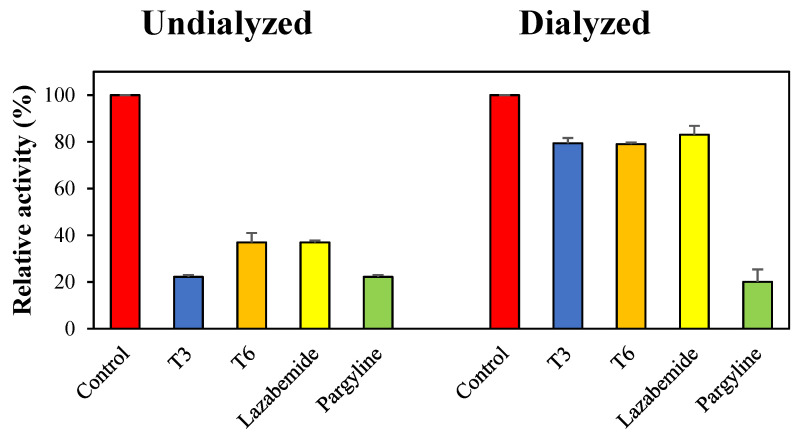
Recoveries of MAO-B inhibitions by **T3** and **T6** by dialysis.

Reversibility studies were performed using a dialysis-based method. Dialysis recovered MAO-B inhibition by **T3** from 22.2% (A_U_) to 79.4% (A_D_) (Figure 3). **T6** was also recovered from 36.9% (A_U_) to 79.0% (A_D_) (Figure 3). These degrees of recovery were similar to that observed for lazabemide (the reversible reference MAO-B inhibitor) (from 36.9% to 83.0%). However, inhibition by pargyline (irreversible MAO-B inhibitor) was not recovered. These experiments showed that inhibitions of MAO-B by **T3** and **T6** were recovered by dialysis to the reversible reference level, thus showing that **T3** and **T6** reversibly inhibit MAO-B.

### 2.5. Cytotoxic Effects of the Active Pyridazinone Derivatives

**Figure 4:**
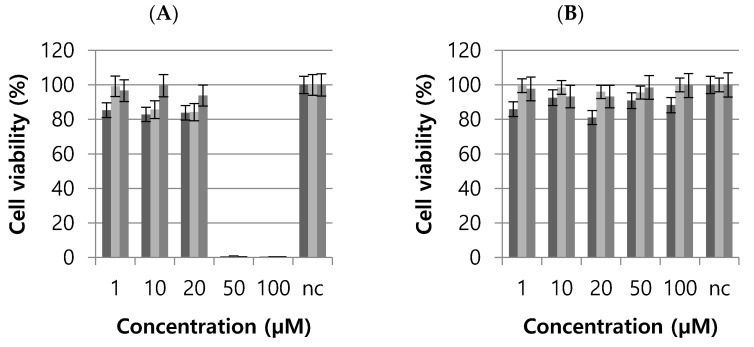
L929 cell viabilities after treatments with **T3** (**A**) or **T6** (**B**). nc, normal control. The experiments were performed with three sets at each concentration.

The cytotoxic effects of **T3** and **T6** were investigated using L929 cells (a healthy fibroblast cell line). **T3** caused complete cell death at 50 and 100 µM), but no significant death at 1, 10, or 20 µM (Figure 4). **T6** did not have a cytotoxic effect at any dose (Figure 4). The IC_50_ values of **T3** and **T6** were 27.05 and 120.6 µM, respectively. These results suggested that **T6** is a more appropriate drug candidate [30].

### 2.6. Molecular Docking Studies

**Figure 5:**
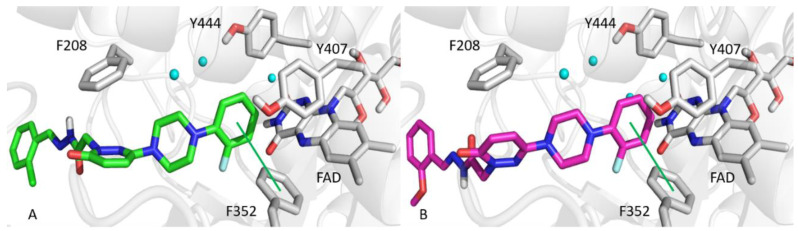
Top scored poses of **T3** (**A**, green sticks) and **T6** (**B**, magenta sticks) towards MAO-A. Green lines show π-π interactions. Water molecules are rendered as cyan spheres.

**Figure 6:**
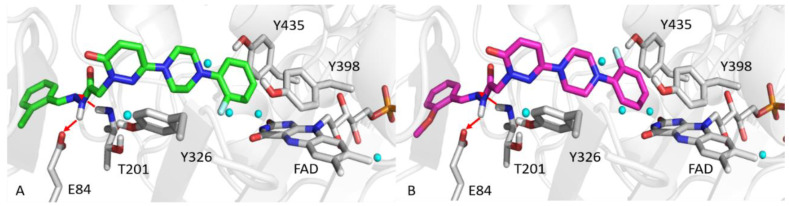
Top scored poses of **T3** (**A**, green sticks) and **T6** (**B**, magenta sticks) towards MAO-B. Red arrows indicate hydrogen bonds. Water molecules are rendered as cyan spheres.

Docking simulations were used to investigate molecular interactions and binding modes of **T3** or **T6** with MAO-A or MAO-B. Simulations docking scores are provided in Table 3. The *ortho*-fluorine phenyl rings of **T3** and **T6** interacted with F352 of MAO-A by π-π contact and by hydrophobic interaction with Y444, Y407, and the FAD of MAO-A (Figure 5). Moreover, the pyridazinone ring of **T6** was well oriented towards F208 of MAO-A, and established another hydrophobic interaction. The *ortho* positioned fluorine on the phenyl ring of **T3** or **T6** faced a hydrophobic cage consisting of FAD, Y435, and Y398 of MAO-B, similar to MAO-A binding mode (Figure 6). Pyridazinone rings participated in hydrophobic interactions with the MAO-B selective residue Y326, and the hydrazone moieties of **T3** and **T6** formed hydrogen bonds with the E84 side-chain and the T201 backbone of MAO-B.

Computational studies of **T3** or **T6** interactions with MAO-A or MAO-B provided satisfactory explanations for the experimental biological data obtained. Docking scores of **T3** and **T6** agreed with IC_50_ experimental values, which indicated **T6** is better than **T3**. Regarding binding modes, the *ortho*-fluorine phenyl rings of **T3** and **T6** face the hydrophobic cages of MAO-A and MAO-B. Interestingly, the main difference between MAO-A and MAO-B interactions centers on the presence of two hydrogen bonds between the hydrazone moieties of **T3** and **T6** and E84 and T201 of MAO-B, due to no hydrogen bond with their corresponding sites V93 and V210 in MAO-A, thus supporting that **T3** and **T6** are selective MAO-B inhibitors.

### 2.7. ADME Prediction

**Figure 7:**
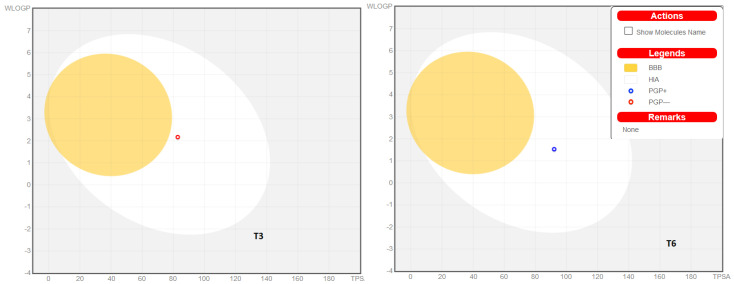
The BOILED-Egg construction of **T3** and **T6**.

**Figure 8:**
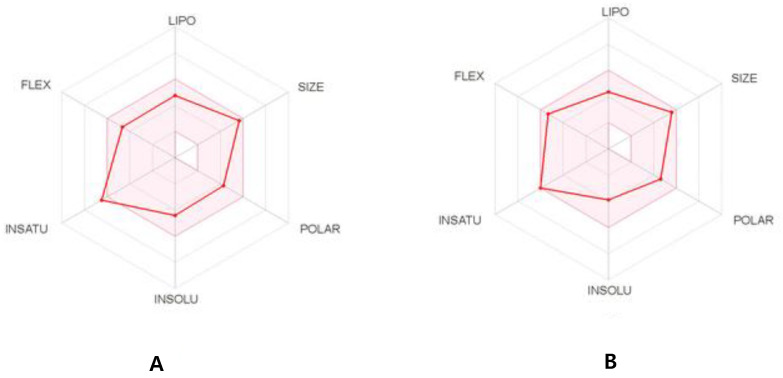
The bioavailability radar of **T3** (**A**) and **T6** (**B**).

The physicochemical parameters and likenesses of **T3** and **T6** were further investigated by BOILED-Egg construction and using the SwissADME online platform. These online programs determine whether compounds are suitable for oral administration based on the bioavailability radar panel containing physicochemical properties such as flexibility, insolubility, lipophilicity, saturation, size, and polarity, and provide rapid appraisals of the drug-likenesses of drug candidates. The BOILED-Egg constructions of **T3** and **T6** predicted that both are well absorbed in the GI tract but in outside of blood-brain barrier (BBB) permeation range (Figure 7). **T3** and **T6** were shown to lie within the pink regions (optimal ranges) (Figure 8). Bioavailability radar predicted both compounds had acceptable pharmacokinetics and satisfied drug-likeness rules. These results suggest that **T3** and **T6** should be considered potential drug candidates.

## 3. Materials and Methods

### 3.1. Chemical Studies

All chemicals used in this study were purchased from Aldrich, Fluka AG, or E. Merck. 3-Chloro-6-substituted pyridazines, 3-chloro-6-(4-(2-fluorophenyl)piperazine-1-yl)pyridazine, 6-(4-(2-fluorophenyl)piperazine-1-yl)-3(*2H*)-pyridazinone, ethyl 6-(4-(2-fluorophenyl)piperazine-1-yl)-3(*2H*)-pyridazinone-2-yl acetate, and 6-(4-(2-fluorophenyl) piperazine-1-yl)-3(*2H*)-pyridazinone-2-yl acetohydrazide were synthesized as previously described [20,21,22,23,24]. The purities of these compounds were checked by TLC using Merck Kieselgel F_254_ plates. Melting points were determined using an Electrothermal 9200 (Cole-Parmer Instrument Co. Vernon Hills, IL, USA), and IR spectra were recorded on a Perkin Elmer Spectrometer using the ATR technique. ^1^H-NMR and ^13^C-NMR spectra were recorded on a Brucker Avonce Ultrashield FT-NMR spectrometer in DMSO at 600 and 75 MHz, respectively. Mass spectra (HRMS) were obtained using a combined Waters Acquity Ultra Performance Liquid Chromatography Micromass and an LCT Premier XE UPLC/MS TOFF spectrophotometer (Waters Corp, Milford, MA, USA) in ESI+ and ESI− modes.

### 3.2. Synthesis of Intermediates

#### 3.2.1. Synthesis of 3-chloro-6-[4-(2-fluorophenyl)piperazine-1-yl]pyridazine (**1**)

A solution containing 0.01 mol of 3,6-dichloropyridazine and 0.01 mol of (2-fluorophenyl)piperazine in 15 mL of ethanol was stirred under reflux at 120 °C for 6 h with the cooler adjusted to 15 °C as previously described [36]. The reaction mix was poured into ice water, and the precipitate obtained by filtration was purified by crystallization from ethanol. Melting points of non-original compounds were in accord with previously reported values [36].

#### 3.2.2. Synthesis of 6-(4-(2-fluorophenyl)piperazine-1-yl)-3(2H)-pyridazinone (**2**)

A solution of 0.05 mol of 3-chloro-6-[4-(2-fluorophenyl)piperazine-1-yl]pyridazine in 30 mL glacial acetic acid was refluxed for 6 h. The acetic acid was then removed under reduced pressure, and the residue obtained was dissolved in water and extracted with chloroform. The organic phase was dried over sodium sulfate and evaporated under reduced pressure, and the residue was purified by recrystallization from ethanol. Melting points of non-original compounds were in accord with previously reported values [36].

#### 3.2.3. Synthesis of ethyl 6-(4-(2-fluorophenyl)piperazine-1-yl)-3(2H)-pyridazinone-2-yl-acetate (**3**)

A mixture of 0.01 mol 6-(4-(2-fluorophenyl)piperazine-1-yl)-3(*2H*)-pyridazinone, 0.02 mol ethyl bromoacetate, and 0.02 mol potassium carbonate in acetone (40 mL) was refluxed overnight. After the mixture had cooled, organic salts were filtered off, the solvent was evaporated, and the residue was purified by recrystallization from n-hexane to give the esters. Melting points of non-original compounds were in accord with previously reported values [36].

#### 3.2.4. Synthesis of 6-(4-(2-fluorophenyl)piperazine-1-yl)-3(2*H*)-pyridazinone-2-yl-acetohydrazide (**4**)

Hydrazine hydrate (99%, 3 mL) was added to the solution of 0.01 mol ethyl 6-(4-(2-fluorophenyl)piperazine-1-yl)-3(2*H*)-pyridazinone-2-yl-acetate in 25 mL methanol and stirred for 3 h at room temperature. The precipitate obtained was filtered off, washed with water, dried, and recrystallized from ethanol. Melting points of non-original compounds were in accord with previously reported values [36].

#### 3.2.5. General Procedure for the Synthesis of **T1**–**T12**

A solution containing 0.01 mol 6-(4-(2-fluorophenyl)piperazine-1-yl)-3(*2H*)-pyridazinone-2-yl-acetohydrazide and 0.01 mol substituted/non-substituted benzaldehyde was stirred in ethanol (15 mL), refluxed for 6 h, and poured into ice water. The precipitate was filtered, dried, and crystallized from methanol:water (8:2, *v*/*v*) [34]. All spectral data of the five new compounds (**T8**–**T12**) were in accord with assigned structures.

#### 3.2.6. 6-[4-(2-Fluorophenyl)piperazine-1-yl]-3(2*H*)-pyridazinone-2-acetyl-2-(3-bromobenzal)hydrazon (**T8**)

White crystals (methanol/water); yield 90.08%; m.p. 207–208 °C; ^1^H-NMR (DMSO-*d*_6_, 300 MHz): δ 3.18 (4H; t; CH_2_N; b+b′), 3.45 (4H; t; CH_2_N; a+a′), 5.31 (2H; s; CH_2_CO), 6.96 (1H; d; *J* = 4.14 Hz, pyridazinone H^5^), 6.97–7.87 (9H; m; phenyl protons, pyridazinone H^4^), 8.58 (1H; s; -N=CH-) and 10.80 (1H; s; -NH-N). ^13^C-NMR (DMSO-*d*_6_, 300 MHz), δ 46.82 (2C, CH_2_-N; b+b′), 50.05 (2C, CH_2_-N; a+a′), 53.14 (1C; -N-CH_2_-C=O), 115.13 (1C; =CH), 119.10 (1C; pyridazinone C^5^), 122.96 (1C; 2-fluorophenyl C^4^), 124.55 (1C; 3-bromophenyl C^5^), 125.90 (1C; 2-fluorophenyl C^5^), 126.05 (1C; 3-bromophenyl C^2^), 129.72 (1C; 3-bromophenyl C^6^), 130.26 (1C; pyridazinone C^4^), 131.08 (1C; 2-fluorophenyl C^6^), 134.22 (1C; 3-bromophenyl C^3^), 142.99 (1C; 2-fluorophenyl C^3^), 147.39 (1C; 3-bromophenyl C^1^), 149.13 (1C; 2-fluorophenyl C^1^), 154.40 (1C; 3-bromophenyl C^4^), 156.56 (1C; pyridazinone C^6^), 158.53 (1C; 2-fluorophenyl C^2^), 161.02 (1C; CH_2_-N-C=O), 168.75 (1C; pyridazinone C^3^); C_23_H_22_BrFN_6_O_2_ MS (ESI+) calculated: 513.3714, found: *m*/*e* 513.10526 (M^+^; 100.0%).

#### 3.2.7. 6-[4-(2-Fluorophenyl)piperazine-1-yl]-3(2H)-pyridazinone-2-acetyl-2-(2-fluorobenzal)hydrazon (**T9**)

White crystals (methanol/water); yield 88.36%; m.p. 225–226 °C; ^1^H-NMR (DMSO-*d*_6_, 300 MHz): δ; 3.19 (4H; t; CH_2_N; b+b′), 3.47 (4H; t; CH_2_N; a+a′), 5.31 (2H; s; CH_2_CO), 6.94 (1H; d; *J* = 4.15 Hz, pyridazinone H^5^), 6.95–7.49 (9H; m; phenyl protons, pyridazinone H^4^), 8.96 (1H; s; -N=CH-) and 10.69 (1H; s; -NH-N). ^13^C-NMR (DMSO-*d*_6_, 300 MHz), 46.63 (2C, CH_2_N; b+b′), 50.07 (2C, CH_2_N; a+a′), 53.29 (1C; -N-CH_2_-C=O), 115.83 (1C; =CH), 116.14 (1C; pyridazinone C^5^), 119.06 (1C; 2-fluorophenyl C^6′^), 122.90 (1C; 2-fluorophenyl C^5′^), 124.51 (1C; 2-fluorophenyl C^4′^), 125.84 (1C; 2-fluorophenyl C^5^), 129.07 (1C; 2-fluorophenyl C^4^), 129.15 (1C; pyridazinone C^4^), 129.62 (1C; 2-fluorophenyl C^6^), 129.70 (1C; 2-fluorophenyl C^3^), 129.78 (1C; 2-fluorophenyl C^3′^), 131.09 (1C; 2-fluorophenyl C^1^), 143.43 (1C; 2-fluorophenyl C^1′^), 147.80 (1C; 2-fluorophenyl C^2′^), 149.09 (1C; pyridazinone C^6^), 154.54 (1C; 2-fluorophenyl C^2^), 158.79 (1C; CH_2_-N-C=O) and 168.73 (1C; pyridazinone C^3^); C_23_H_22_F_2_N_6_O_2_ MS (ESI+) calculated: 452.47, found: 453.18594 *m*/*e* (M^+^; 100.0%).

#### 3.2.8. 6-[4-(2-Fluorophenyl)piperazine-1-yl]-3(2*H*)-pyridazinone-2-acetyl-2-(4-florobenzal)hydrazon (**T10**)

White crystals (methanol/water); yield 57.70%; m.p. 178–180 °C; ^1^H-NMR (DMSO-*d*_6_, 300 MHz): δ; 3.17 (4H; t; CH_2_N; b+b′), 3.46 (4H; t; CH_2_N; a+a′), 5.30 (2H; s; CH_2_CO), 6.84 (1H; d; *J* = 4.15 Hz, pyridazinone H^5^), 6.85–7.83 (9H; m; phenyl protons, pyridazinone H^4^), 8.08 (1H; s; -N=CH-) and 10.68 (1H; s; -NH-N). ^13^C-NMR (DMSO-*d*_6_, 300 MHz), 46.84 (2C, CH_2_N; b+b′), 50.07 (2C, CH_2_N; a+a′), 53.29 (1C; -N-CH_2_-C=O), 115.83 (1C; =CH), 116.05 (1C; pyridazinone C^5^), 116.35 (1C; 4-fluorophenyl C^6^), 122.90 (1C; 4-fluorophenyl C^5^), 124.51 (1C; 4-fluorophenyl C^4^), 125.84 (1C; 2-fluorophenyl C^5^), 129.07 (1C; 2-fluorophenyl C^4^), 129.62 (1C; pyridazinone C^4^), 129.70 (1C; 2-fluorophenyl C^6^), 130.68 (1C; 2-fluorophenyl C^3^), 139.71 (1C; 4-fluorophenyl C^3^), 147.80 (1C; 2-fluorophenyl C^1^), 149.09 (1C; 4-fluorophenyl C^1^), 154.54 (1C; 4-fluorophenyl C^2^), 158.79 (1C; pyridazinone C^6^), 162.52 (1C; 2-fluorophenyl C^2^), 165.16 (1C; CH_2_-N-C=O) and 168.73 (1C; pyridazinone C^3^); C_23_H_22_F_2_N_6_O_2_ MS (ESI+) calculated: 452.47, found: 453.18583 *m*/*e* (M^+^; 100.0%).

#### 3.2.9. 6-[4-(2-Fluorophenyl)piperazine-1-yl]-3(2*H*)-pyridazinone-2-acetyl-2-(4-methoxybenzal)hydrazon (**T11**)

White crystals (methanol/water); yield 75.32%; m.p. 148–150 °C. ^1^H-NMR (DMSO-*d*_6_, 300 MHz): δ 3.17 (4H; t; CH_2_N; b+b′), 3.45 (4H; t; CH_2_N; a+a′), 3.85 (3H; s; CH_3_O), 5.30 (2H; s; CH_2_CO), 6.88 (1H; d; *J* = 4.15 Hz, pyridazinone H^5^), 6.89–7.79 (9H; m; phenyl protons, pyridazinone H^4^), 8.64 (1H; s; -N=CH-) and 10.55 (1H; s; -NH-N). ^13^C-NMR (DMSO-*d_6_*, 300 MHz), 46.83 (2C, CH_2_N; b+b′), 50.07 (2C, CH_2_N; a+a′), 53.03 (1C; -N-CH_2_-C=O), 55.38 (1C; -OCH_3_), 114.07 (1C; =CH), 116.12 (1C; pyridazinone C^5^), 119.07 (1C; 4-methoxyphenyl C^6^), 124.50 (1C; 4-methoxyphenyl C^5^), 125.77 (1C; 4-methoxyphenyl C^4^), 126.30 (1C; 2-fluorophenyl C^5^), 128.82 (1C; 2-fluorophenyl C^4^), 129.43 (1C; pyridazinone C^4^), 130.14 (1C; 2-fluorophenyl C^6^), 131.10 (1C; 2-fluorophenyl C^3^), 139.63 (1C; 4-methoxyphenyl C^3^), 144.42 (1C; 2-fluorophenyl C^1^), 149.04 (1C; 4-methoxyphenyl C^1^), 154.39 (1C; 4-methoxyphenyl C^2^), 156.83 (1C; pyridazinone C^6^), 158.82 (1C; 2-fluorophenyl C^2^), 161.35 (1C; CH_2_-N-C=O) and 168.56 (1C; pyridazinone C^3^); C_24_H_25_FN_6_O_3_ MS (ESI+) calculated: 464.49, found: 465.20506 *m*/*e* (M^+^; 100.0%).

#### 3.2.10. 6-[4-(2-Fluorophenyl)piperazine-1-yl]-3(2H)-pyridazinone-2-acetyl-2-(2-methylbenzal)hydrazon (**T12**)

White crystals (methanol/water); yield 72.21%; m.p. 244–245 °C; ^1^H-NMR (DMSO-*d*_6_, 300 MHz): δ 2.50 (3H; s; -CH_3_), 3.17 (4H; t; CH_2_N; b+b′), 3.45 (4H; t; CH_2_N; a+a′), 5.30 (2H; s; CH_2_CO), 6.92 (1H; d; *J* = 4.19 Hz, pyridazinone H^5^), 6.92–7.77 (9H; m; phenyl protons, pyridazinone H^4^), 8.37 (1H; s; -N=CH-) and 10.58 (1H; s; -NH-N). ^13^C-NMR (DMSO-*d_6_*, 300 MHz), δ 20.16 (1C; -CH_3_), 46.69 (2C; CH_2_-N; b+b′), 50.10 (2C, CH_2_-N; a+a′), 53.36 (1C; -N-CH_2_-C=O), 116.13 (1C; =CH), 119.07 (1C; pyridazinone C^5^), 122.87 (1C; 4-methylphenyl C^6^), 124.54 (1C; 2-methylphenyl C^2^), 125.78 (1C; 2-methylphenyl C^3^), 126.24 (1C; 4-methylphenyl C^5^), 127.47 (1C; 4-methylphenyl C^4^), 129.96 (1C; pyridazinone C^4^), 130.85 (1C; 2-fluorophenyl C^3^), 131.48 (1C; 2-fluorophenyl C^4^), 137.13 (1C; 2-fluorophenyl C^5^), 143.70 (1C; 2-methylphenyl C^1^), 147.49 (1C; 2-fluorophenyl C^6^), 149.71 (1C; 2-fluorophenyl C^1^), 156.98 (1C; 2-fluorophenyl C^2^), 158.81 (1C; pyridazinone C^6^) 163.96 (1C; CH_2_-N-C=O) and 168.57 (1C; pyridazinone C^3^); C_24_H_25_FN_6_O_2_ MS (ESI+) calculated: 448.49, found: 449.21034 *m*/*e* (M^+^; 100.0%).

### 3.3. Enzyme Assays

Recombinant human MAO-A and MAO-B inhibitory activities of were continuously assayed as described previously [37], using kynuramine (0.06 mM) and benzylamine (0.3 mM), respectively. Substrate concentrations were 1.5 × and 2.3 × K_m_, respectively, and their K_m_ values were 0.041 mM and 0.13 mM, respectively. MAO-A and MAO-B inhibitory activities were measured after preincubating enzymes and inhibitors for 15 min. Enzymes and chemicals were obtained from Sigma-Aldrich (St. Louis, MO, USA).

### 3.4. Enzyme Inhibitions and Kinetics

The inhibitory activities of the twelve compounds against MAO-A and MAO-B were first investigated at an inhibitor concentration of 10 µM. For the compounds showing residual activities of <50%, IC_50_ values for MAO-A and MAO-B inhibitions were determined. Kinetic experiments were carried out at five substrate and three inhibitor concentrations [38].

### 3.5. Analysis of Inhibitor Reversibilities

The reversibilities of **T3** and **T6** inhibitions were investigated by dialysis after preincubating them with MAO-B for 30 min, as previously described [39]. The concentrations used were **T3** 0.080 µM, **T6** 0.020 µM, lazabemide (reference reversible MAO-B inhibitor) 0.30 µM, and pargyline (reference irreversible MAO-B inhibitor) 0.060 µM. Reversibilities were determined using the relative activities of undialyzed (A_U_) and dialyzed (A_D_) samples [40].

### 3.6. Cytotoxicity

An MTS (5-(3-carboxymethoxyphenyl)-2-(4,5-dimethyl-thiazolyl)-3-(4-sulfophenyl)) tetrazolium assay was used to evaluate the cytotoxic activities of **T3** and **T6** by measuring formazan absorbance. MTS is converted to formazan by mitochondria in metabolically active cells via NAD(P)H-dependent dehydrogenase enzymes. L929 cells (a mouse fibroblast cell line) were used to evaluate the cytotoxicities. Briefly, cells (5 × 10^4^ cells/well) were seeded into 96-well plates in DMEM containing 10% fetal bovine serum and 1% penicillin/streptomycin, and incubated for 24 h. After supernatants were removed, the cells were treated with the compounds dissolved in DMSO (at 1, 10, 20, 50, or 100 µM) in triplicate for 48 h at 37 °C in a 5% CO_2_ atmosphere. MTS reagent (Uptima, Interchim, Montluçon, France) was then added to each well and incubated for 3 h. Absorbances were measured at 490 nm using a microplate reader (BioTek, Winooski, VT, USA), and cell viabilities were calculated as percentage of untreated controls [30].

### 3.7. Molecular Docking Studies

**T3** and **T6** were docked using X-ray solved structures of MAO-A and MAO-B obtained from the Protein Data Bank (PDB) by retrieving entries 2Z5X and 2V5Z, respectively [41,42]. Protein targets were prepared using the Protein Preparation Wizard Schrödinger Suite 2018-4 (New York, NY, USA) for refining and optimizing crystal structures after correcting for protonation states and adding missing hydrogen atoms [43,44]. Nine water molecules within MAO-A and eight water molecules within MAO-B were not deleted for docking simulations, as was previously performed [45]. To generate all possible tautomers and protonation states at physiological pH, **T3** and **T6** were treated using the LigPrep tool [46]. The obtained files were used for docking simulations by employing Grid-based Ligand Docking with Energetics (GLIDE) [47]. Enclosing boxes were automatically centered on the related cognate ligands, **T3** and **T6**. The default force Field OPLS_20058 and the standard precision (SP) docking protocol with default settings were employed for the in silico analyses. To confirm the validity of docking studies, re-docking simulations were performed on **T3** and **T6** at their binding sites. The cognate ligands are flagged as HRM and SAG for MAO-A and MAO-B, respectively, and moved back to the original positions with Root Mean Square Deviations (RMSD) accounting for all heavy atoms equal to 0.760 Å and 0.395 Å for HRM and SAG, respectively [48].

### 3.8. ADME Prediction

ADME parameters were predicted in silico to determine a range of physical-chemical properties, lipophilicity, water solubility, and pharmacokinetic data, using the login-free website http://www.swissadme.ch/ [49].

## 4. Conclusions

The present work describes the synthesis of twelve pyridazinones (**T1**–**T12**) containing the 2-fluorophenyl substituted piperazine moiety. All synthesized compounds were evaluated for MAO-A and MAO-B inhibitory activities. Most of the compounds were selective MAO-B inhibitors, and **T3** and **T6** were found to be selective, competitive, and reversible MAO-B inhibitors. **T3** and **T6** potently and reversibly inhibited MAO-B in the sub-micromolar range, and were relatively non-toxic to healthy fibroblasts. We concluded that **T3** and **T6** offer new developmental leads for the treatment of neurodegenerative disorders.

## Figures and Tables

**Table 1:** Molecular structures, yields, melting points, molecular weights, and molecular formulae of T1-12.

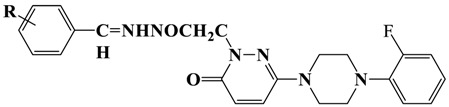					
Compound No.	R	Yield (%)	P	MM	Molecular Formula
**T1**	H	79.90	226–228	434.48	C_23_H_23_FN_6_O_2_
**T2**	4-CH_3_	78.15	198–200	448.49	C_24_H_25_FN_6_O_2_
**T3**	2-Cl	73.19	203–205	468.91	C_23_H_22_ClFN_6_O_2_
**T4**	4-Cl	91.21	192–194	468.91	C_23_H_22_ClFN_6_O_2_
**T5**	4-Br	85.69	209–211	513.36	C_23_H_22_BrFN_6_O_2_
**T6**	2-CH_3_O	77.45	239–241	464.49	C_24_H_25_FN_6_O_3_
**T7**	4-N(CH_3_)_2_	68.77	237–239	477.53	C_25_H_28_FN_7_O_2_
**T8**	3-Br	90.08	207–208	513.36	C_23_H_22_BrFN_6_O_2_
**T9**	2-F	88.36	225–226	452.47	C_23_H_22_ F_2_N_6_O_2_
**T10**	4-F	57.70	178–180	452.47	C_23_H_22_ F_2_N_6_O_2_
**T11**	4- OCH_3_	75.32	148–150	464.49	C_24_H_25_FN_6_O_3_
**T12**	2-CH_3_	72.21	244–245	448.49	C_24_H_25_FN_6_O_2_

**Table 2:** Inhibitions of recombinant human MAO-A and MAO-B by T1 to T12 ^a^.

Compounds	Residual Activity (%)		IC_50_ (µM)		SI ^b^
	MAO-A	MAO-B	MAO-A	MAO-B	
	(10 µM)	(1.0 µM)			
**T1**	96.6 ± 1.20	99.4 ± 0.85	-	7.68 ± 0.14	-
**T2**	24.6 ± 2.86	4.22 ± 0.85	4.29 ± 0.12	0.10 ± 0.035	42.9
**T3**	24.6 ± 0.97	3.01 ± 0.85	4.19 ± 0.27	0.039 ± 0.0028	107.4
**T4**	75.2 ± 4.70	6.00 ± 0.94	-	0.099 ± 0.0069	-
**T5**	84.0 ± 0.68	54.0 ± 2.83	-	1.68 ± 0.11	-
**T6**	20.7 ± 0.62	−15.3 ± 0.94	1.57 ± 0.80	0.013 ± 0.0016	120.8
**T7**	25.4 ± 6.92	57.8 ± 9.58	4.43 ± 0.29	0.87 ± 0.056	-
**T8**	73.3 ± 2.86	67.2 ± 9.58	-	2.67 ± 0.051	-
**T9**	35.2 ± 0.56	2.60 ± 0.74	6.76 ± 0.46	0.20 ± 0.0017	33.8
**T10**	33.7 ± 4.70	95.3 ± 6.70	7.35 ± 0.059	35.3 ± 0.39	0.21
**T11**	70.4 ± 8.50	61.1 ± 2.98	-	6.86 ± 0.37	-
**T12**	61.8 ± 1.65	17.9 ± 5.95	-	0.15 ± 0.021	-
Toloxatone			1.08 ± 0.025	-	
Lazabemide			-	0.14 ± 0.011	
Clorgyline			0.0070 ± 0.00070	-	
Pargyline			-	0.030 ± 0.00083	

^a^ Results are expressed as the means ± standard errors of duplicate or triplicate experiments; ^b^ Selectivity index (SI) values shown are expressed as IC50 of MAO-A/IC50 of MAO-B; IC50 values for reference compounds were determined after preincubating them for 30 min with MAO-A or MAO-B.

**Table 3:** Docking score values for T3 and T6 interactions with MAO-A or MAO-B.

Compounds	Docking Score (kcal/mol)	
	MAO-A	MAO-B
**T3**	−8.20	−9.45
**T6**	−8.43	−9.56

## Supplementary Materials

^1^H- and ^13^C-NMR spectral data are available in the Supplementary Materials.
