# Supplementary material for: Design, Synthesis, and Biological Evaluation of Pyridazinones Containing the (2-Fluorophenyl) Piperazine Moiety as Selective MAO-B Inhibitors
Source: Molecules. 2020 Nov 17;25(22):5371. doi: 10.3390/molecules25225371 (PMC7698448; doi:10.3390/molecules25225371)

Supplementary Materials

Design, Synthesis, and Biological Evaluation of Pyridazinones Containing the (2-Fluorophenyl) Piperazine Moiety as selective MAO-B Inhibitors

Muhammed Çeçen [1,†], Jong Min Oh [2,†], Zeynep Özdemir [1,*], Saliha Ebru Büyüktuncel [3], Mehtap Uysal [4], Mohamed A. Abdelgawad [5,6], Arafa Musa [7,8], Nicola Gambacorta [9], Orazio Nicolotti [9], Bijo Mathew [10,*] and Hoon Kim [2,*]

[1] Department of Pharmaceutical Chemistry, Faculty of Pharmacy, Inonu University, Malatya 44280, Turkey; mcecen2344@gmail.com
[2] Department of Pharmacy, and Research Institute of Life Pharmaceutical Sciences, Sunchon National University, Suncheon 57922, Korea; ddazzo005@naver.com
[3] Department of Analytical Chemistry, Faculty of Pharmacy, Inonu University, Malatya 44280, Turkey; saliha.buyuktuncel@inonu.edu.tr
[4] Department of Pharmaceutical Chemistry, Faculty of Pharmacy, Gazi University, Ankara 06100, Turkey; mgokce99@gmail.com
[5] Department of Pharmaceutical Chemistry, College of Pharmacy, Jouf University, Sakaka, Al Jouf 72341, Saudi Arabia; mohamedabdelwahab976@yahoo.com
[6] Department of Pharmaceutical Organic Chemistry, Faculty of Pharmacy, Beni-Suef University, Beni Suef 62514, Egypt
[7] Department of Pharmacognosy, College of Pharmacy, Jouf University 72341, Sakaka, Saudi Arabia; akmusa@ju.edu.sa
[8] Department of Pharmacognosy, Faculty of Pharmacy, Al-Azhar University, Cairo 11371, Egypt
[9] Dipartimento di Farmacia-Scienze del Farmaco, Università degli Studi di Bari "Aldo Moro", Via E. Orabona, 4, I-70125 Bari, Italy; nicola.gambacorta1@uniba.it (N.G.); orazio.nicolotti@uniba.it (O.N.)
[10] Department of Pharmaceutical Chemistry, Amrita School of Pharmacy, Amrita Vishwa Vidyapeetham, Amrita Health Science Campus, Kochi-682 041, India
* Correspondence: zeynep.bulut@inonu.edu.tr (Z.Ö.); bijovilaventgu@gmail.com (B.M.); hoon@sunchon.ac.kr or hoon@scnu.ac.kr (H.K.)
† These authors contributed equally to this work.

Data 1. ^{1}H-NMR spectrum of compound T1

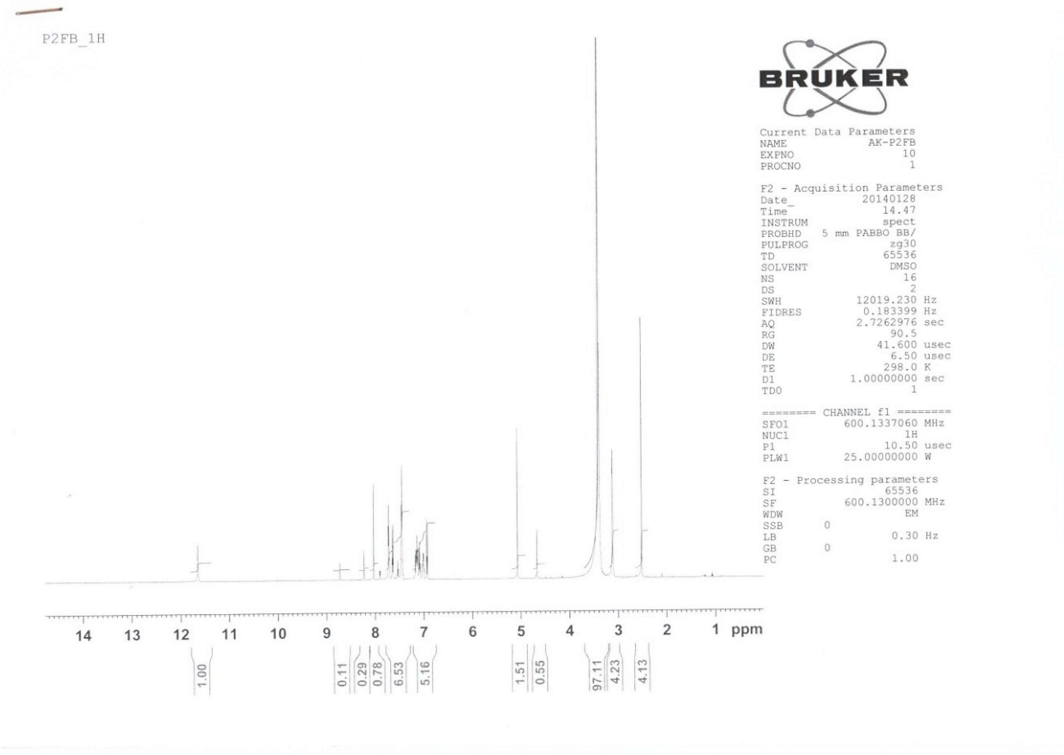

Data 2. ^{13}C-NMR spectrum of compound T1

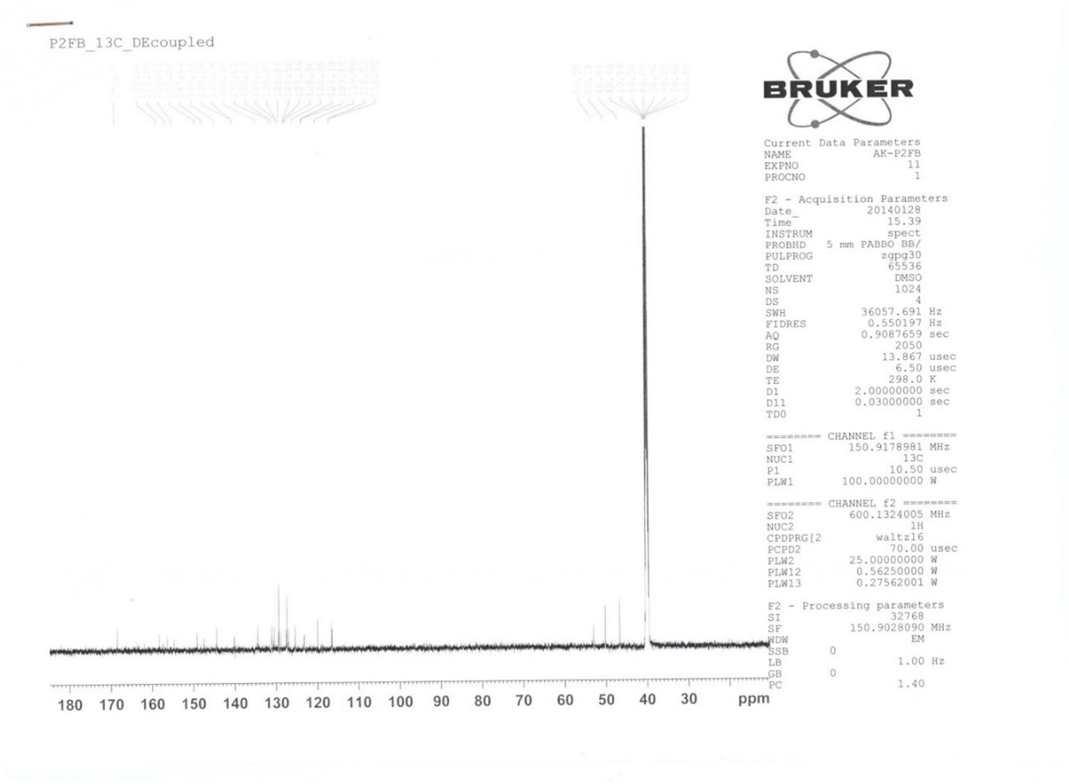

Data 3. HRMS spectrum of compound T1

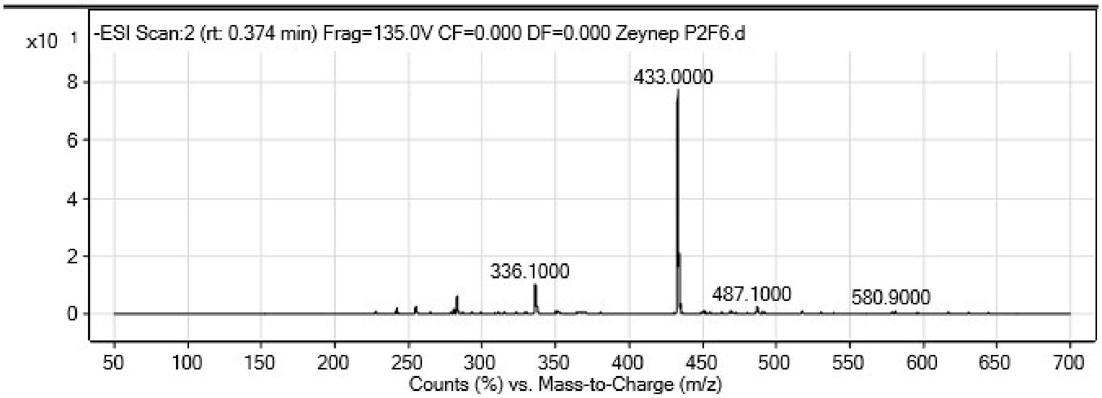

Peak List

m/z	z	Abund
241.9		2196.5
255.1	1	3057.06
280.8		1658.48
283.1		7019.18
336.1	1	11244.46
337.1	1	3218.38
433	1	85811.05
434	1	22975.82
435	1	3972.78

Data 4. ^{1}H-NMR spectrum of compound T2

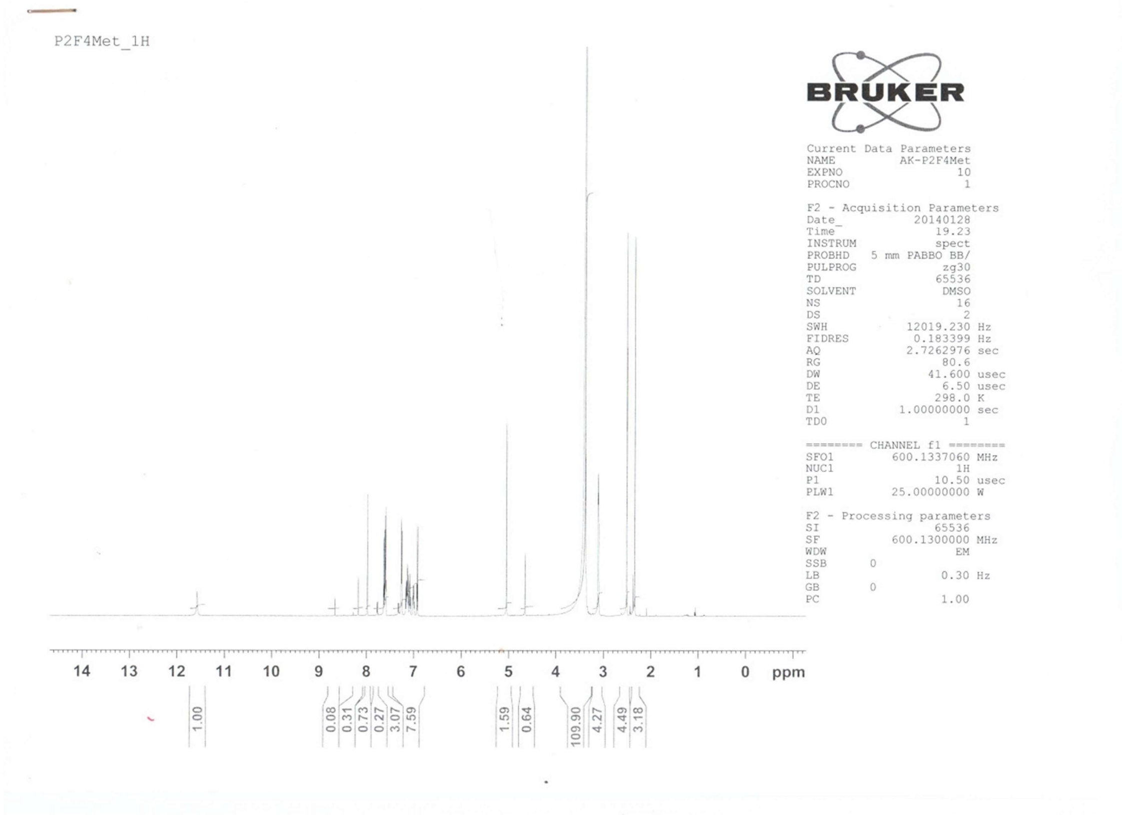

Data 5. ^{13}C-NMR spectrum of compound T2

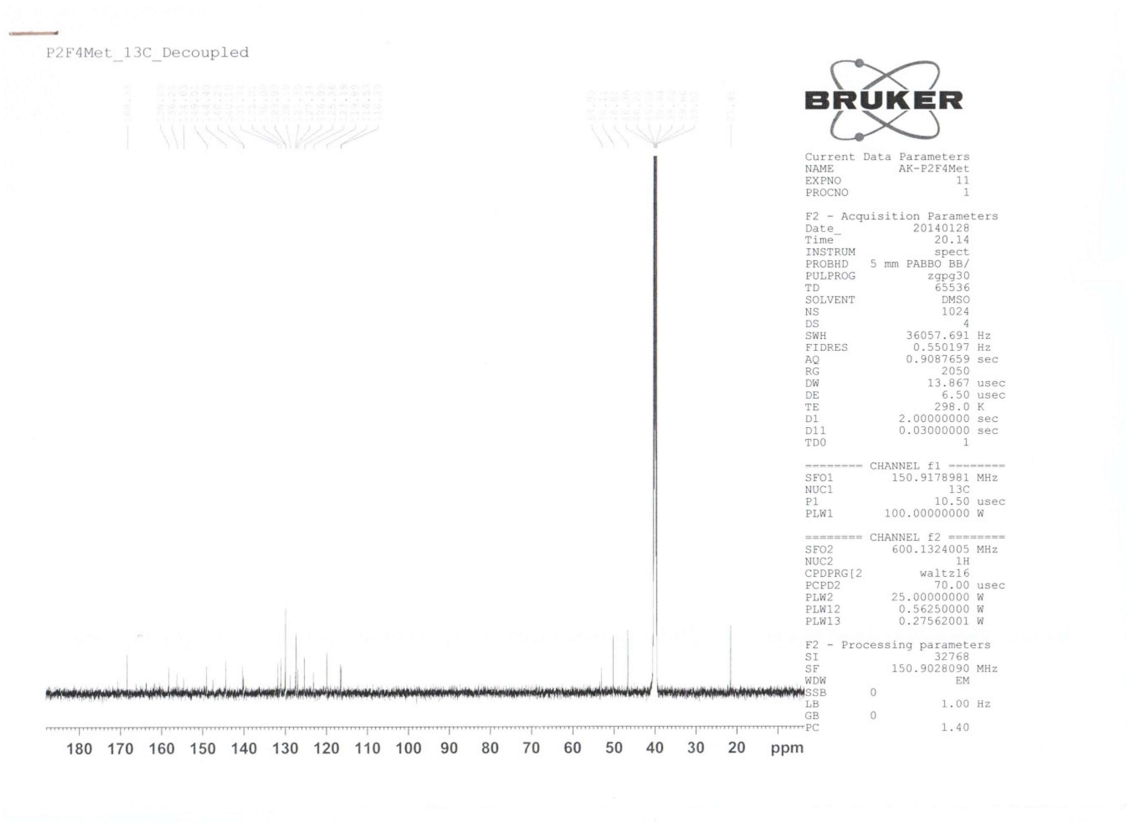

Data 6. HRMS spectrum of compound T2

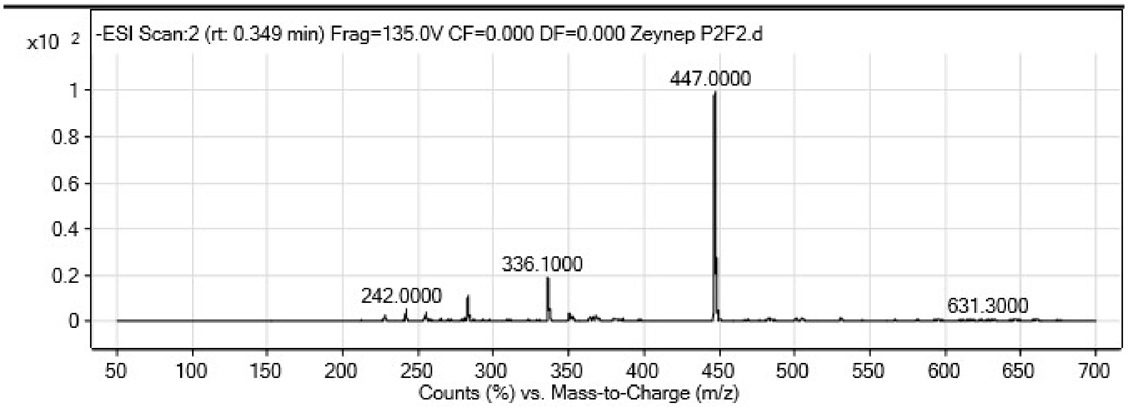

Peak List

m/z	z	Abund
228.1		2618.28
242		5255.52
255.1		3681.6
283		10534.4
336.1		18198.36
337.2	1	4868.92
350.1		3531.82
447	1	93741.25
448	1	25748.56
449	1	4422.98

Data 7. ^{1}H-NMR spectrum of compound T3

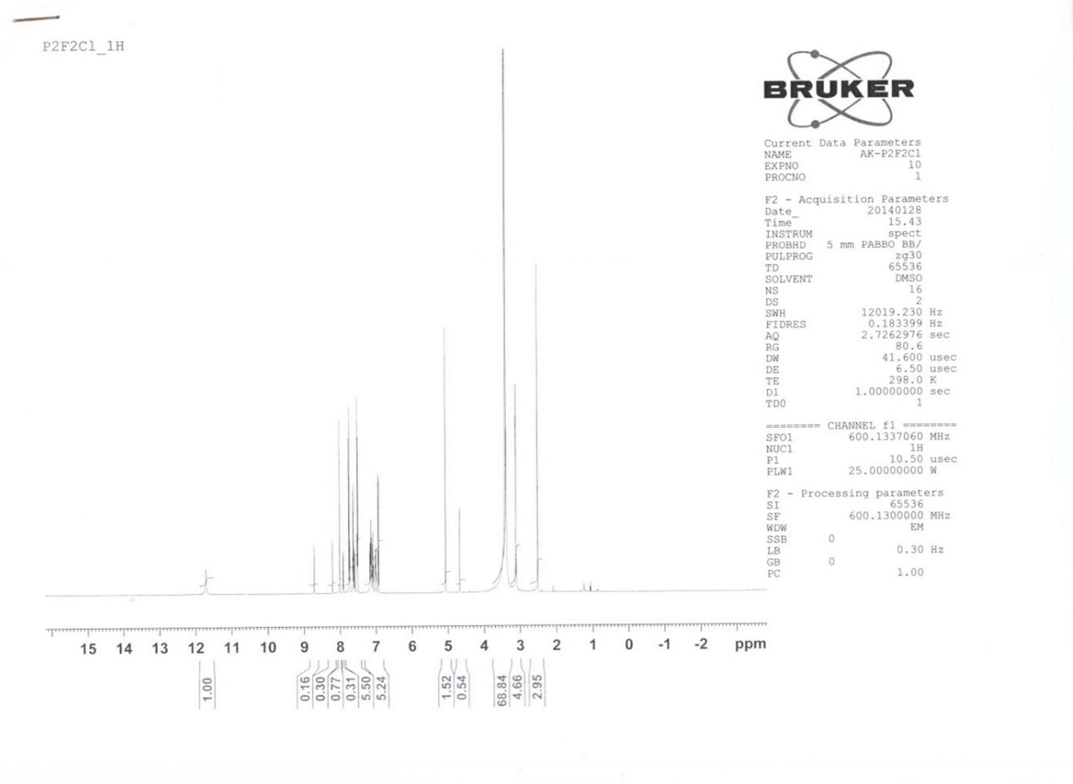

Data 8. ^{13}C-NMR spectrum of compound T3

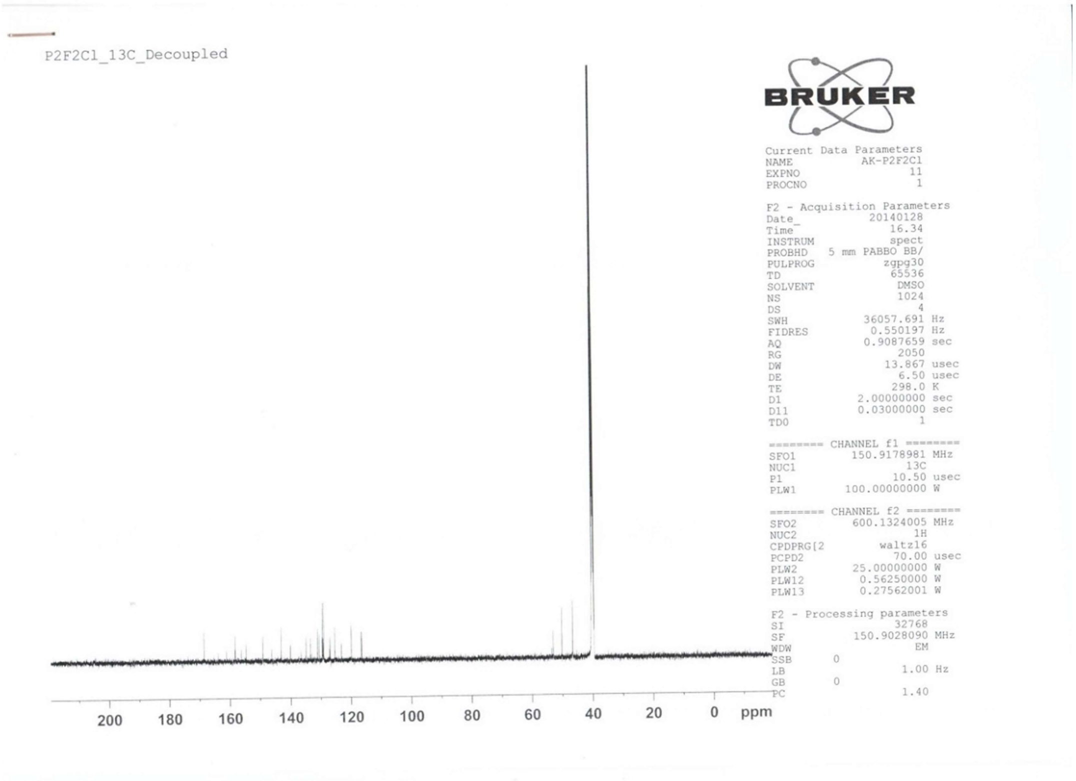

Data 9. HRMS spectrum of compound T3

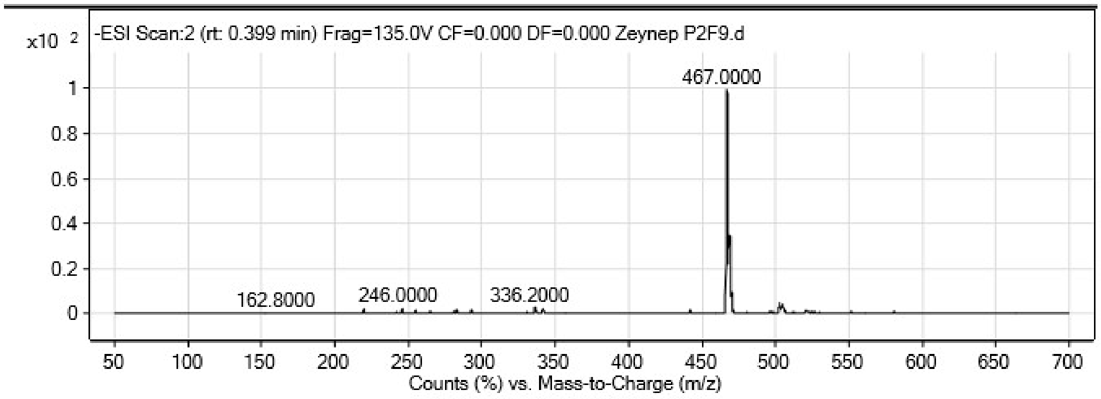

Peak List

m/z	z	Abund
220	1	2460.52
246		2666.44
336.2		3515.12
341.6	2	2662.1
467		118998.15
468.3		40049.68
469	1	41925.7
470	1	11476.82

Data 10. ^{1}H-NMR spectrum of compound T4

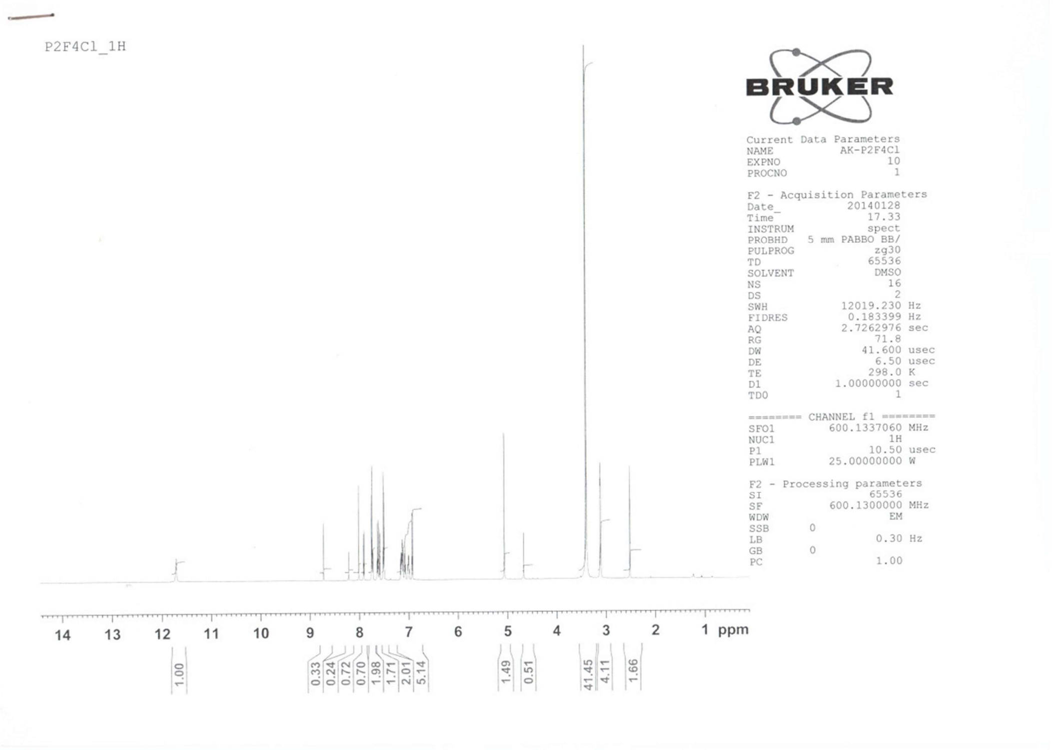

Data 11. ^{13}C-NMR spectrum of compound T4

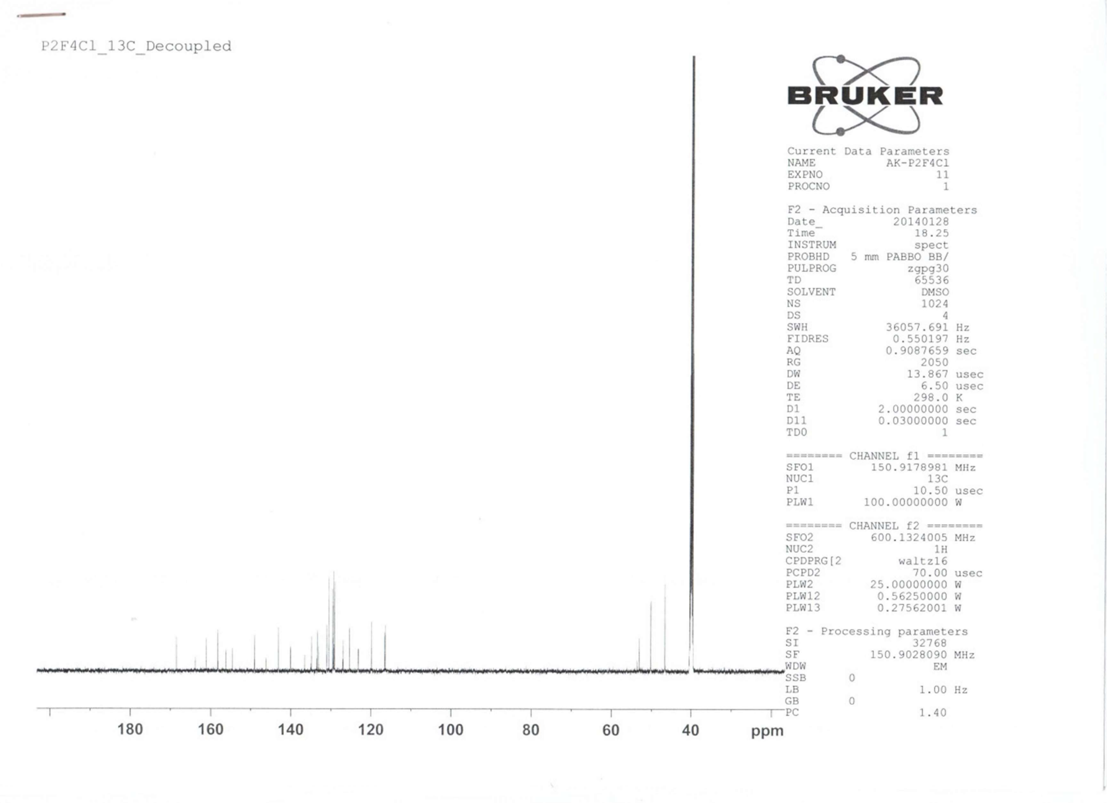

Data 12. HRMS spectrum of compound T4

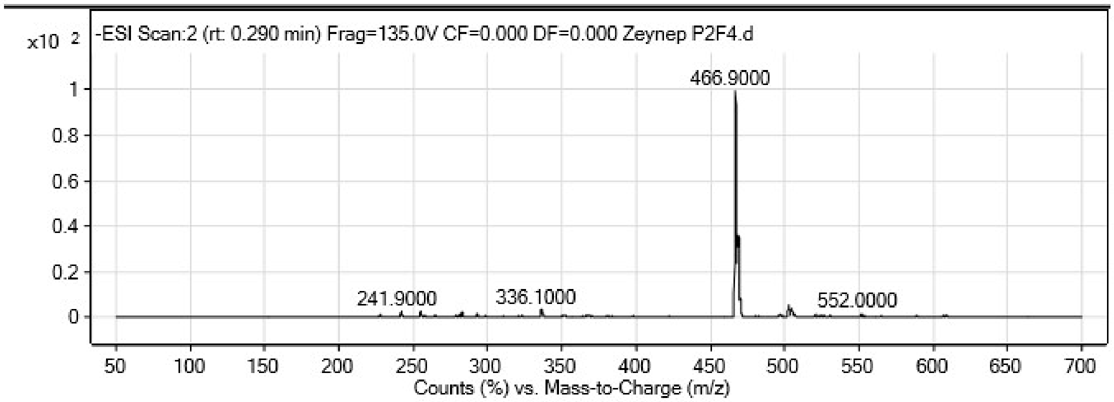

Peak List

m/z	z	Abund
241.9		3589.42
254.7	2	2865.68
255.1		3232.08
336.1	1	4549.26
466.9		121424.79
468.2		43604.76
468.9	1	43965.76
469.9	1	10811.56

Data 13. ^{1}H-NMR spectrum of compound T5

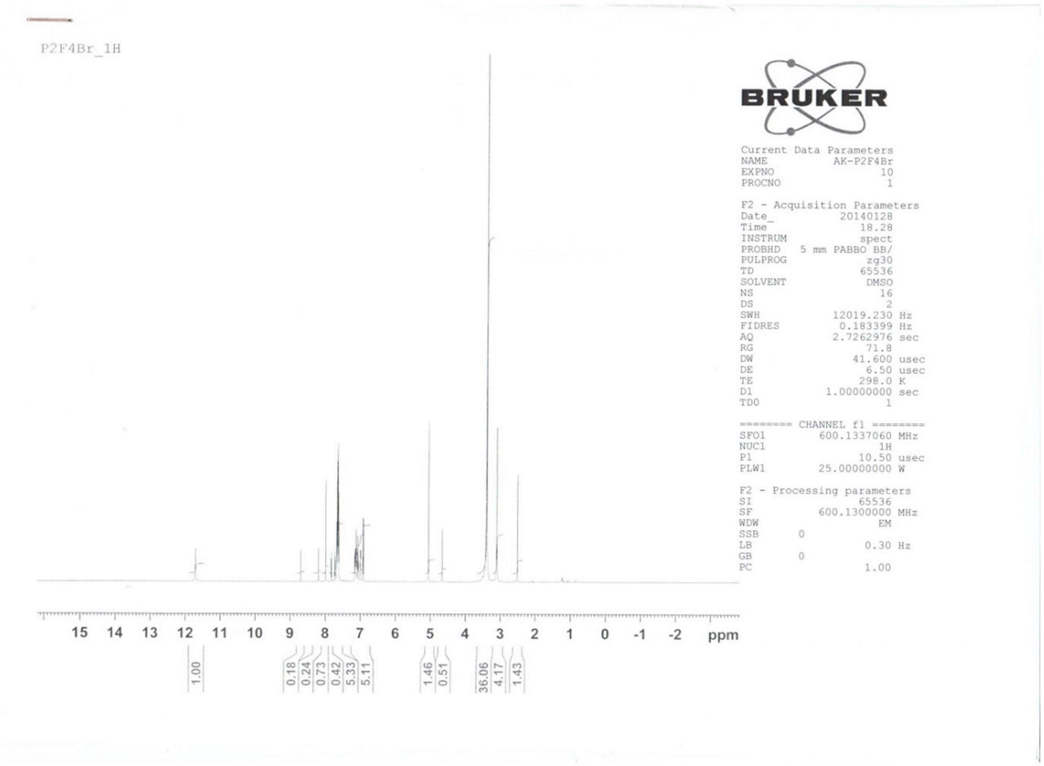

Data 14. ^{13}C-NMR spectrum of compound T5

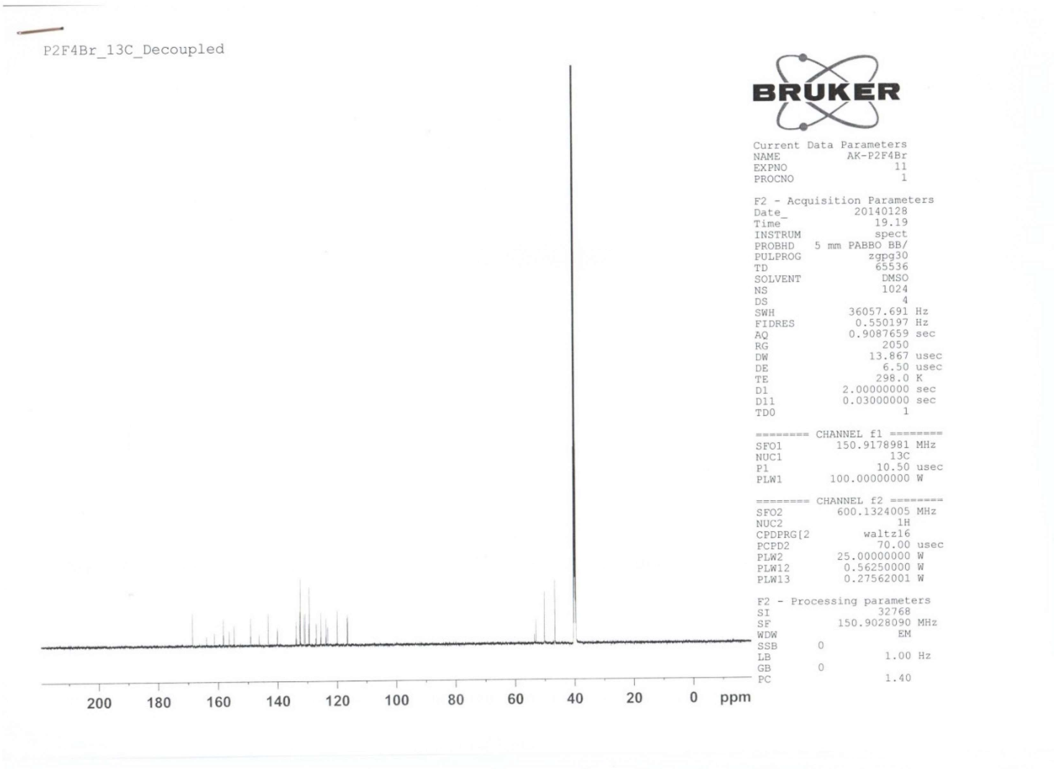

Data 15. HRMS spectrum of compound T5

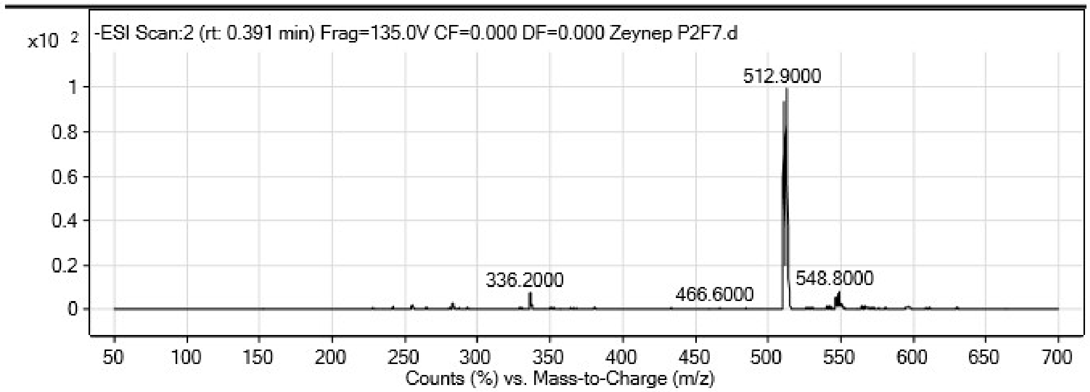

Peak List

m/z	z	Abund
283.1		2781.52
336.2	1	7290.16
510.9		93370.91
512.9	1	99546.02
513.9	1	25357.88
514.9	1	4260.32

Data 16. ¹H-NMR spectrum of compound T6

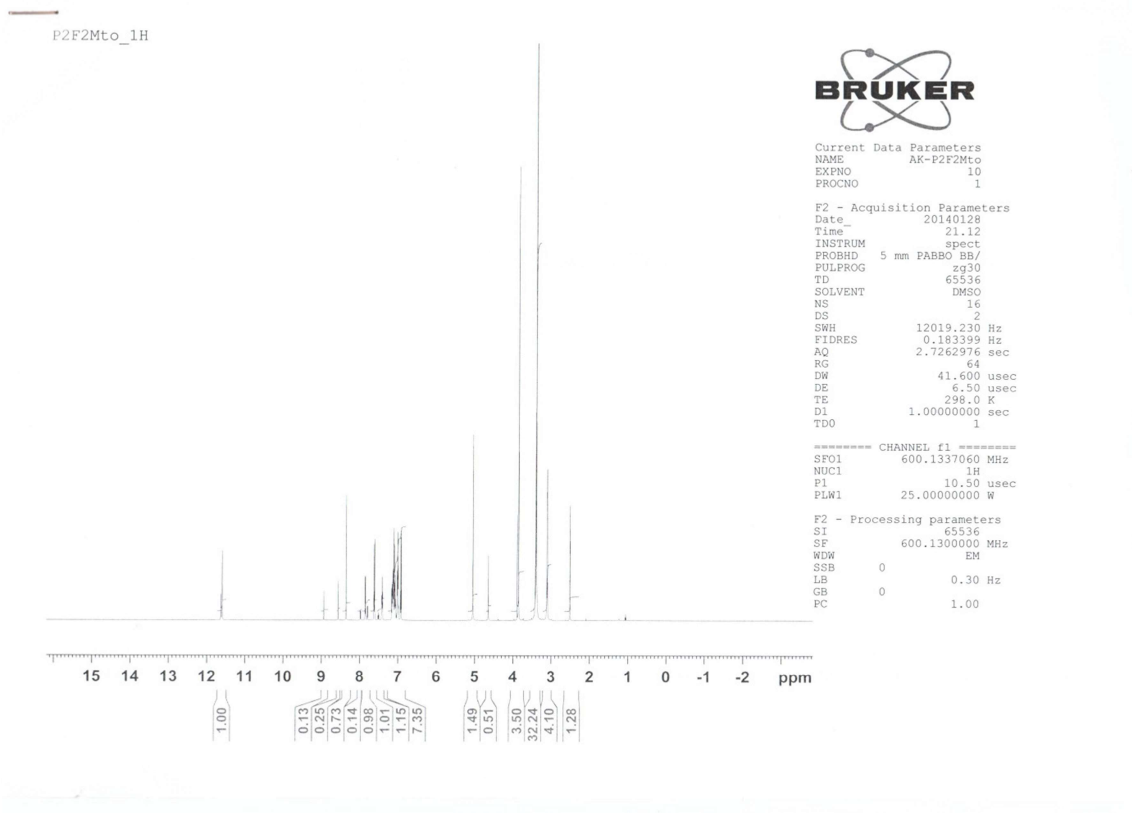

Data 17. ¹³C-NMR spectrum of compound T6

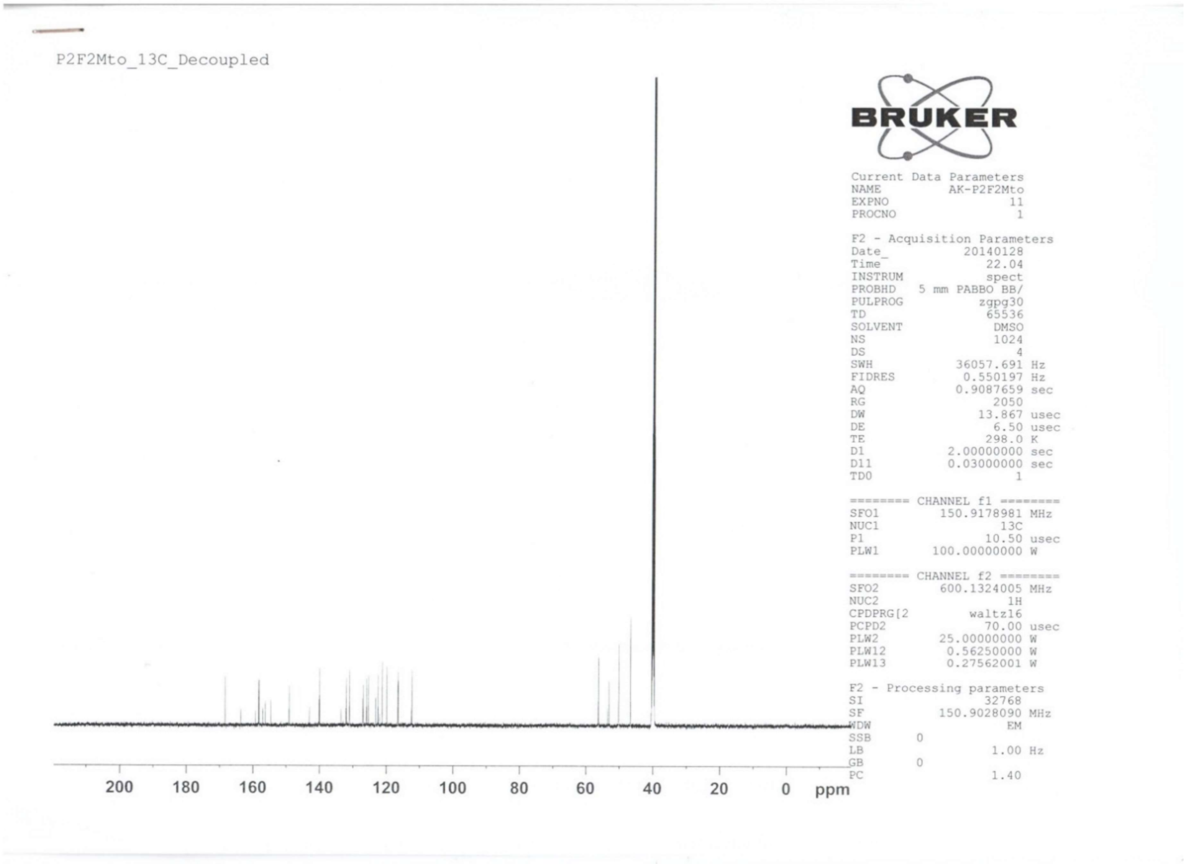

Data 18. HRMS spectrum of compound T6

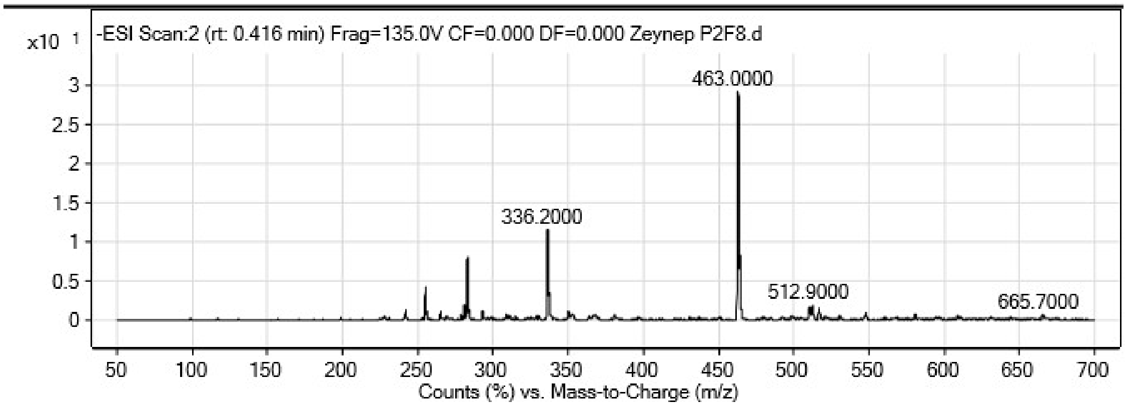

Peak List

m/z	Abund
255.1	4895.28
281	2339.54
283	9136.32
336.2	13092.22
337.1	3950.88
463	32864.1
464.1	9337.26

Data 19. ^{1}H-NMR spectrum of compound T7

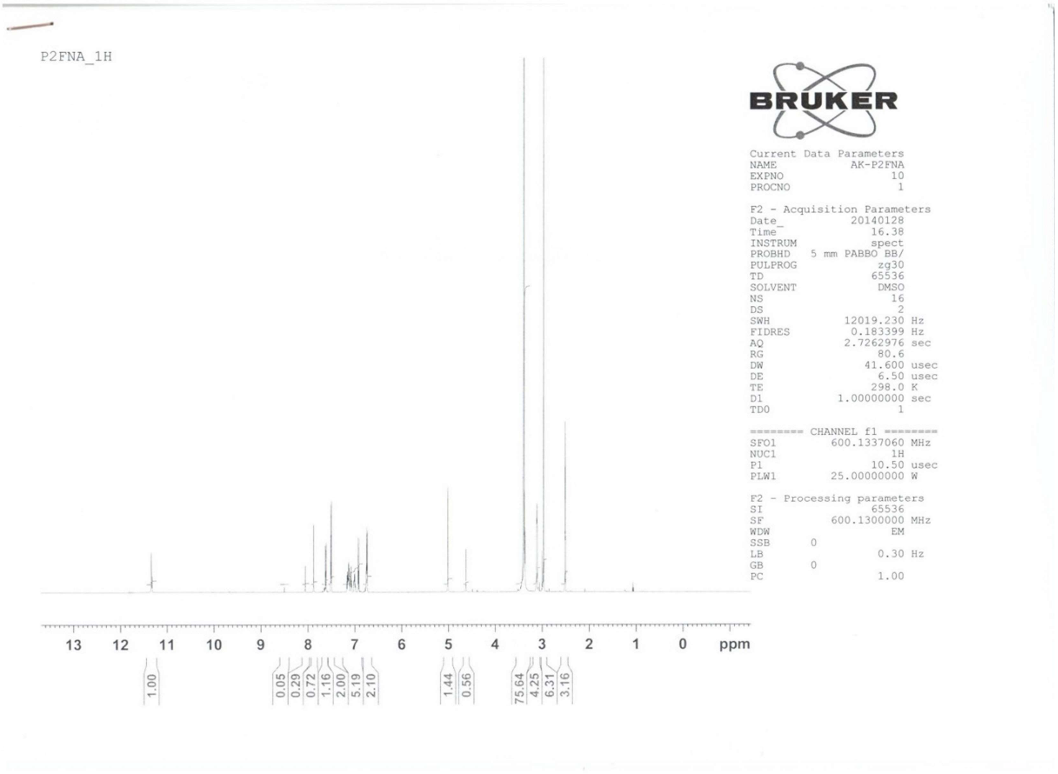

Data 20. ^{13}C-NMR spectrum of compound T7

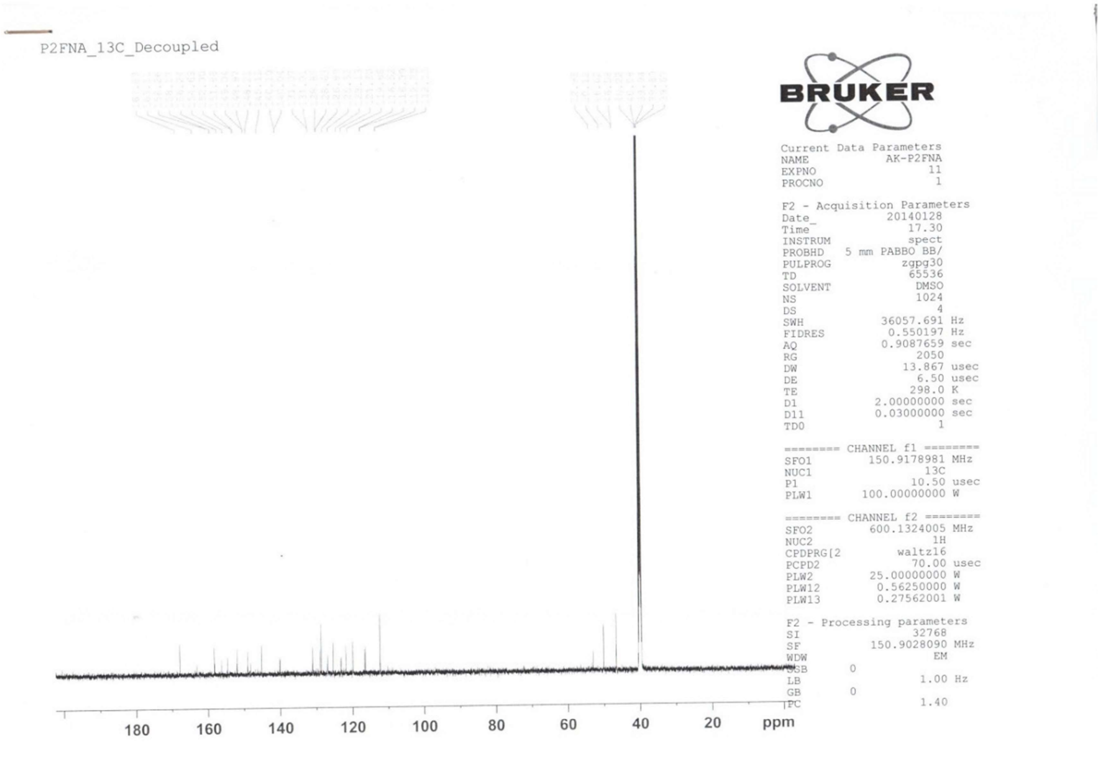

Data 21. HRMS spectrum of compound T7

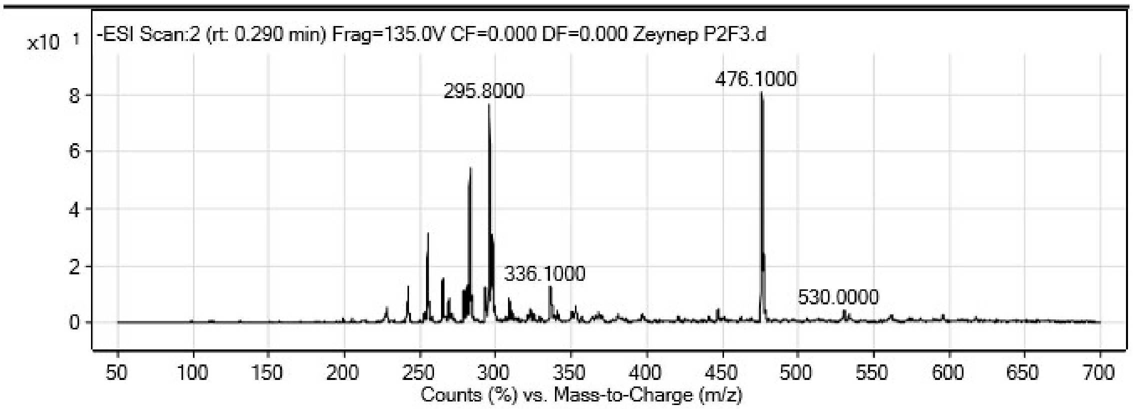

Peak List

m/z	z	Abund
242.1		4177.8
255.1	1	9973.54
264.9	1	4965.84
281.1		4239.44
283	1	17355.72
295.8		24492.76
297.7	2	9943.44
336.1		4078.94
476.1	1	25842.92
477.1	1	7893.2

<New compounds>

Data 1. ^{1}H-NMR spectrum of compound T8

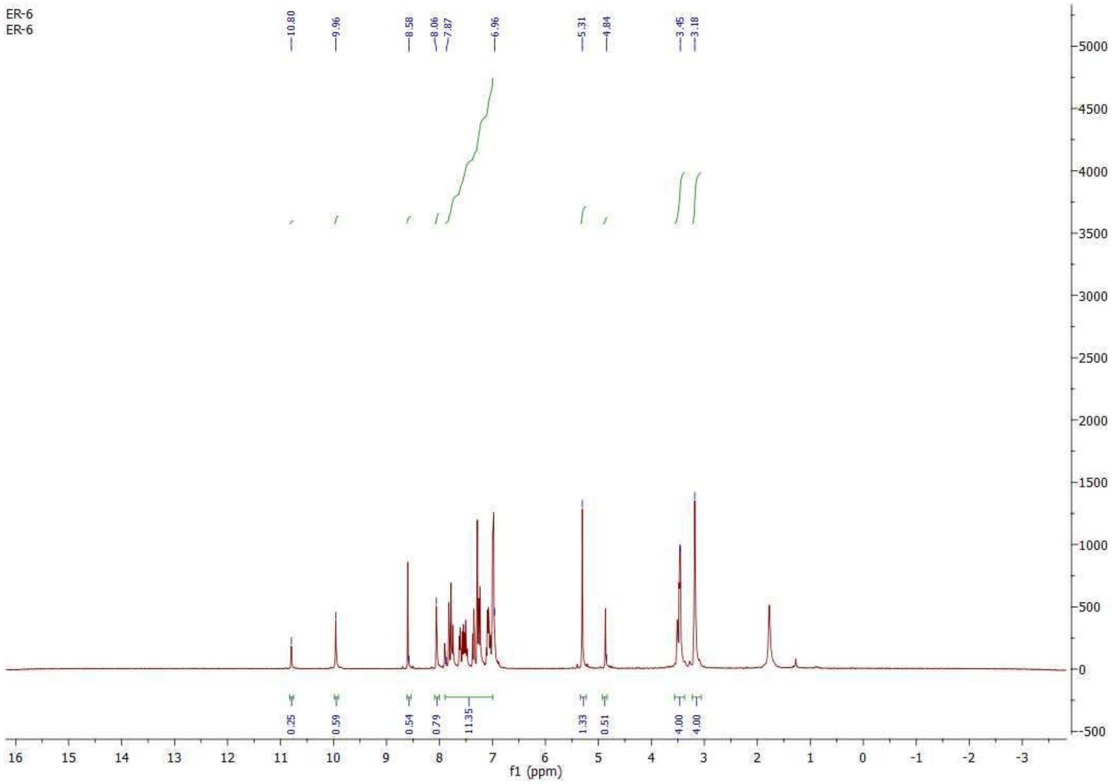

Data 2. ^{13}C-NMR spectrum of compound T8

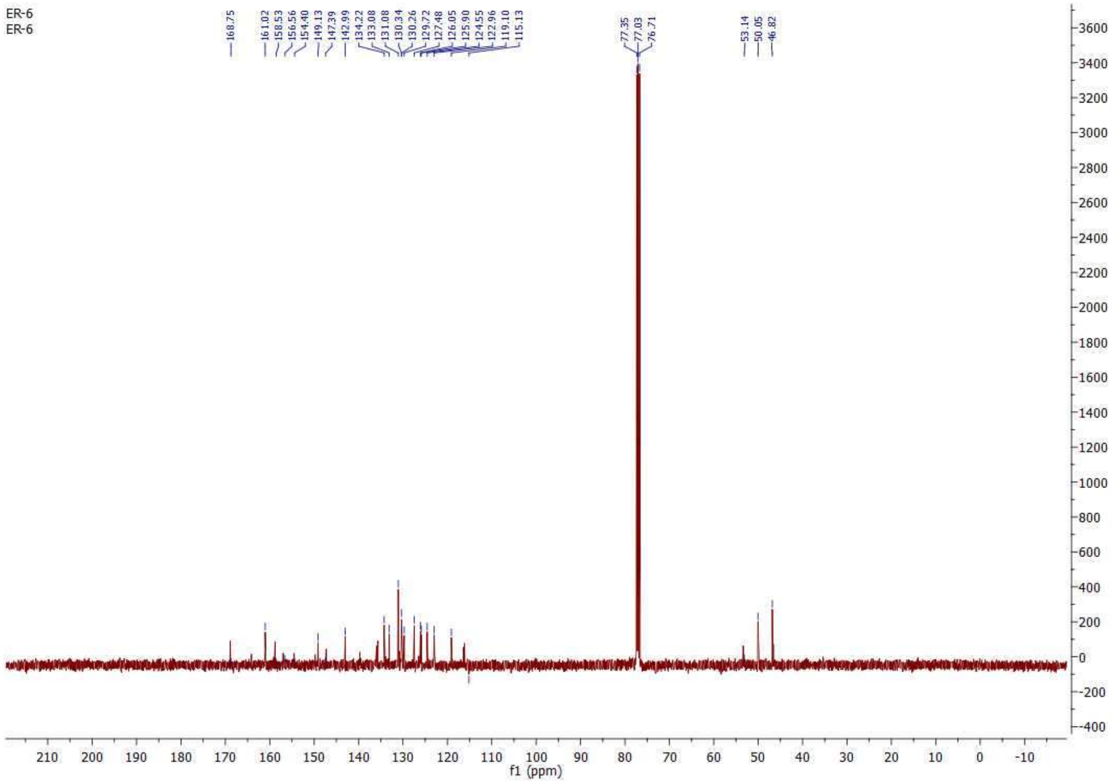

Data 3. HRMS spectrum of compound T8

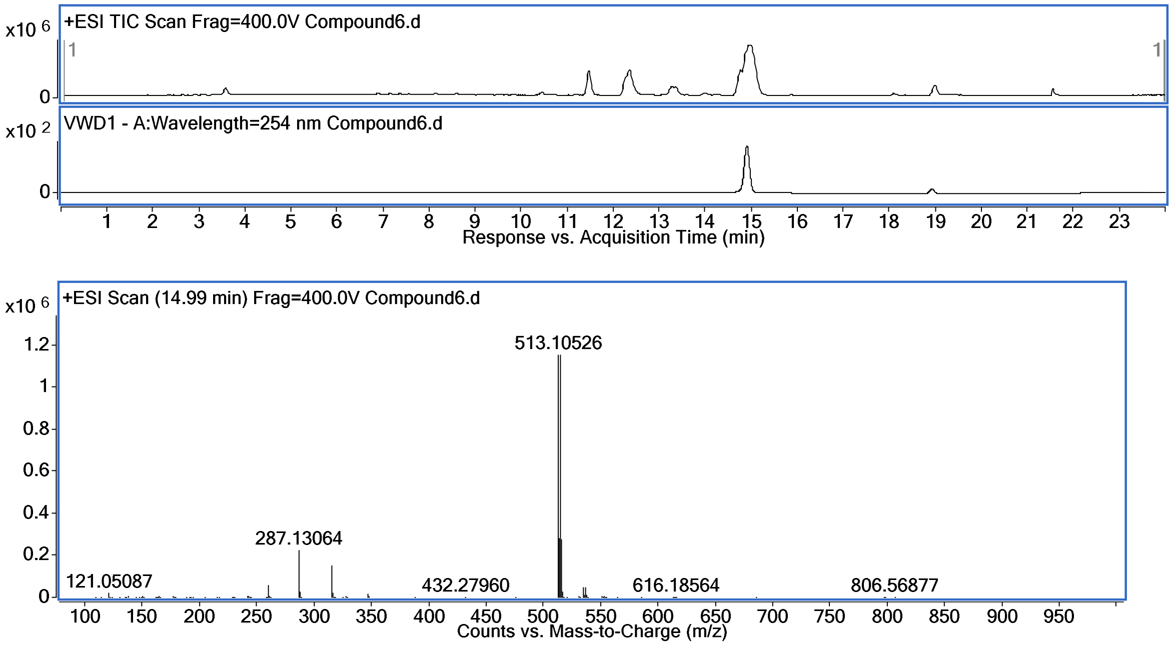

Data 4. ^{1}H-NMR spectrum of compound T9

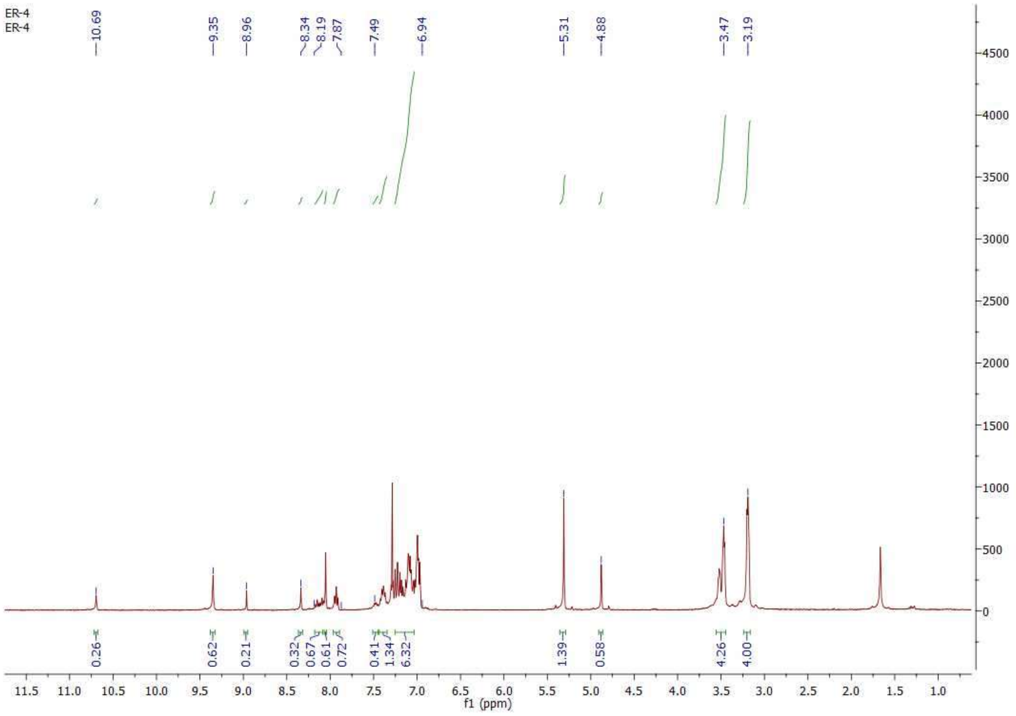

Data 5. ^{13}C-NMR spectrum of compound T9

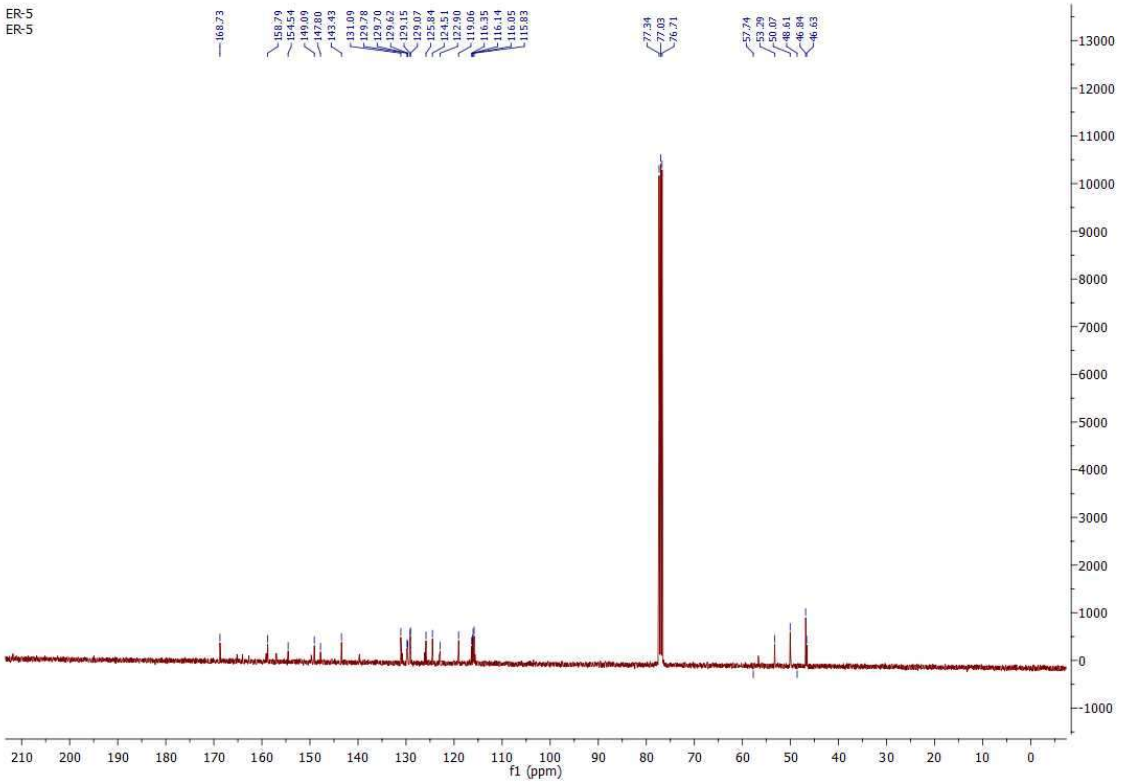

Data 6. HRMS spectrum of compound T9

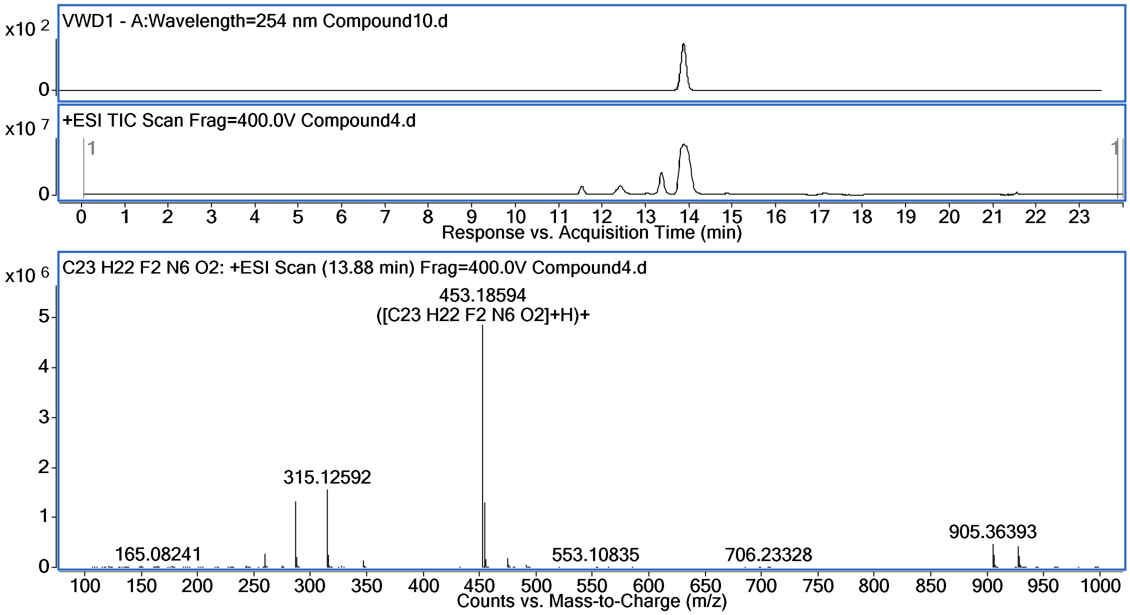

Data 7. ^{1}H-NMR spectrum of compound T10

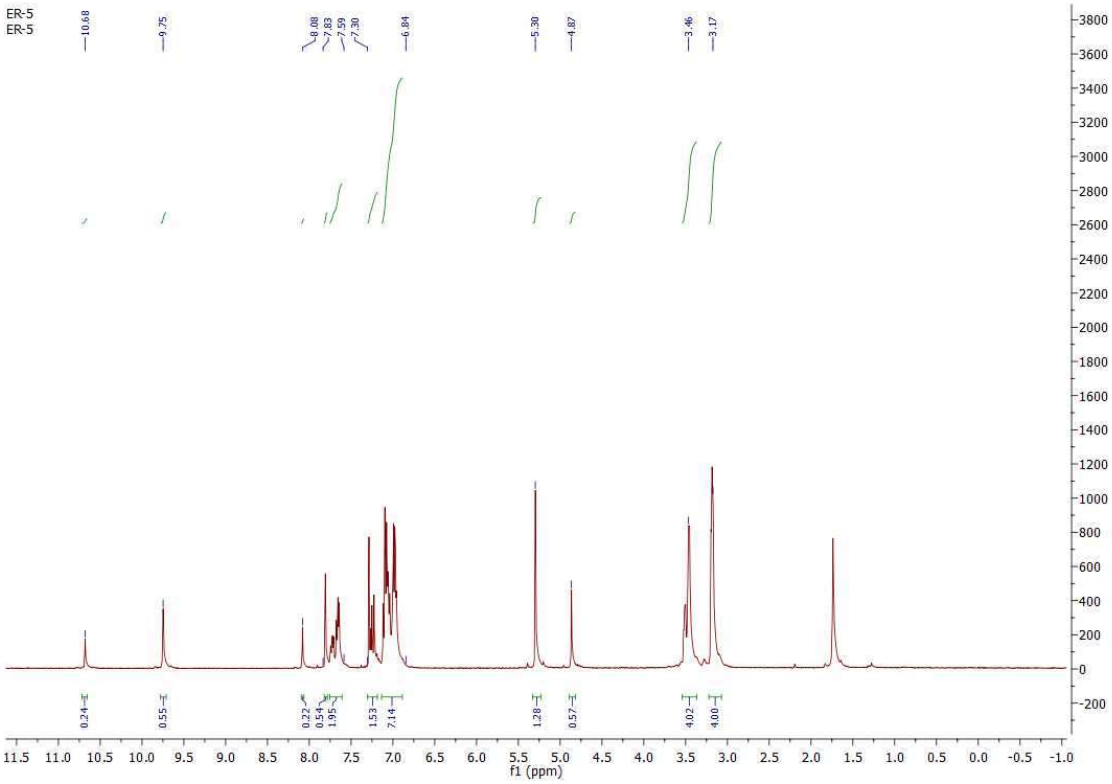

Data 8. ^{13}C-NMR spectrum of compound T10

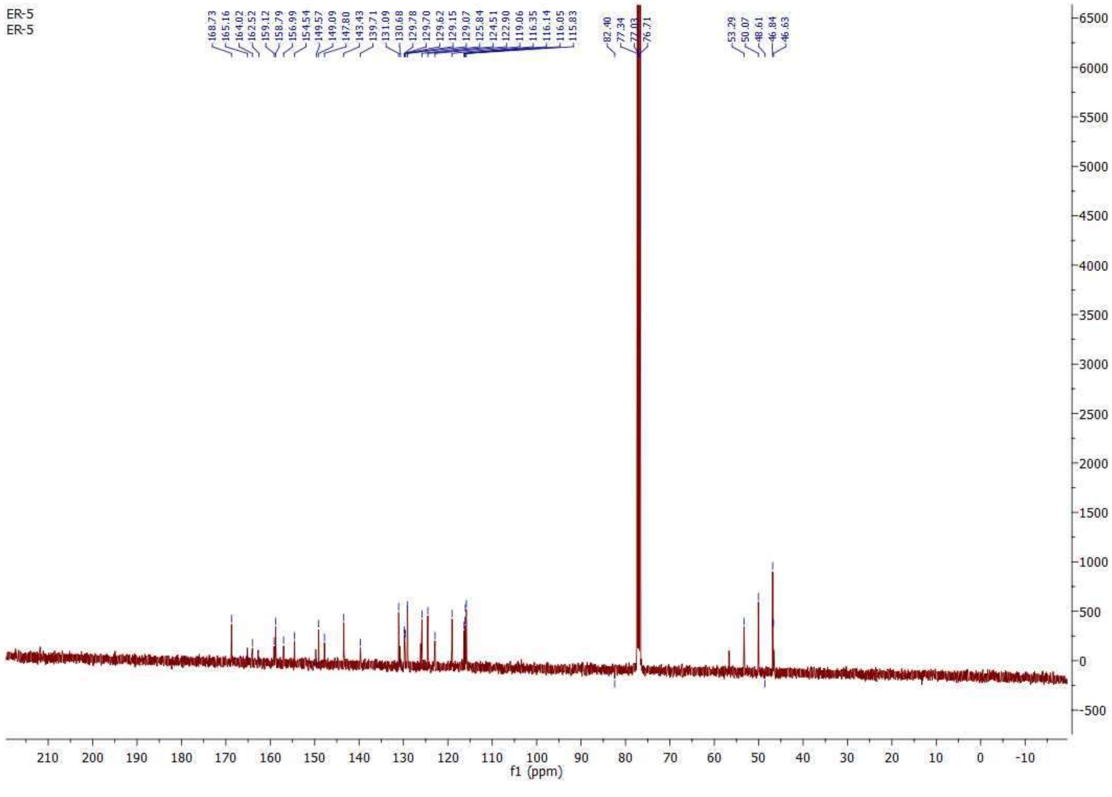

Data 9. HRMS spectrum of compound T10

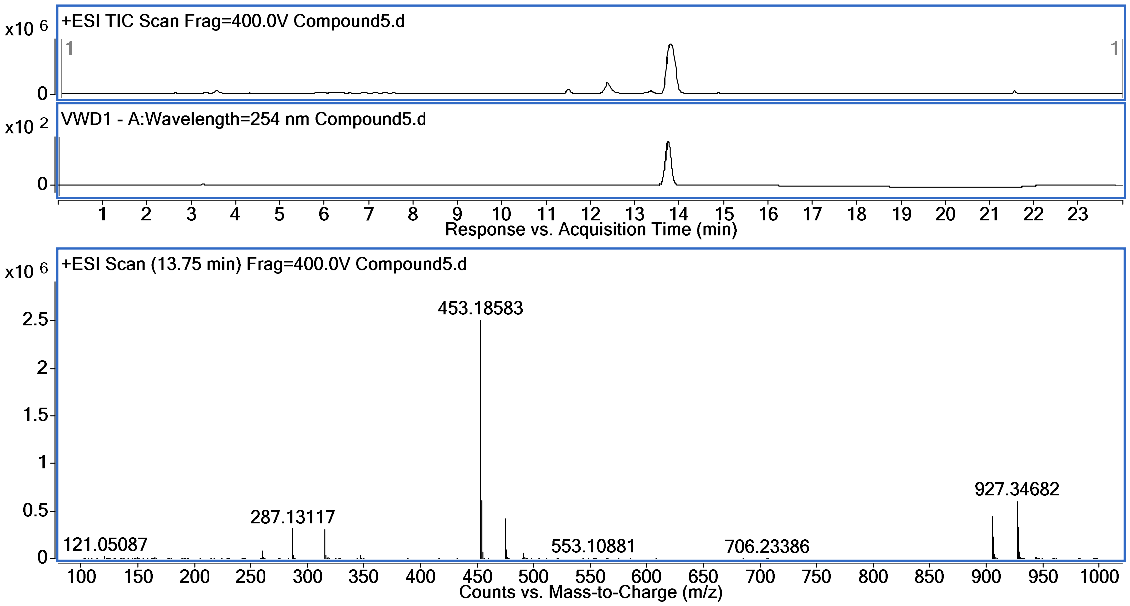

Data 10. ^{1}H-NMR spectrum of compound T11

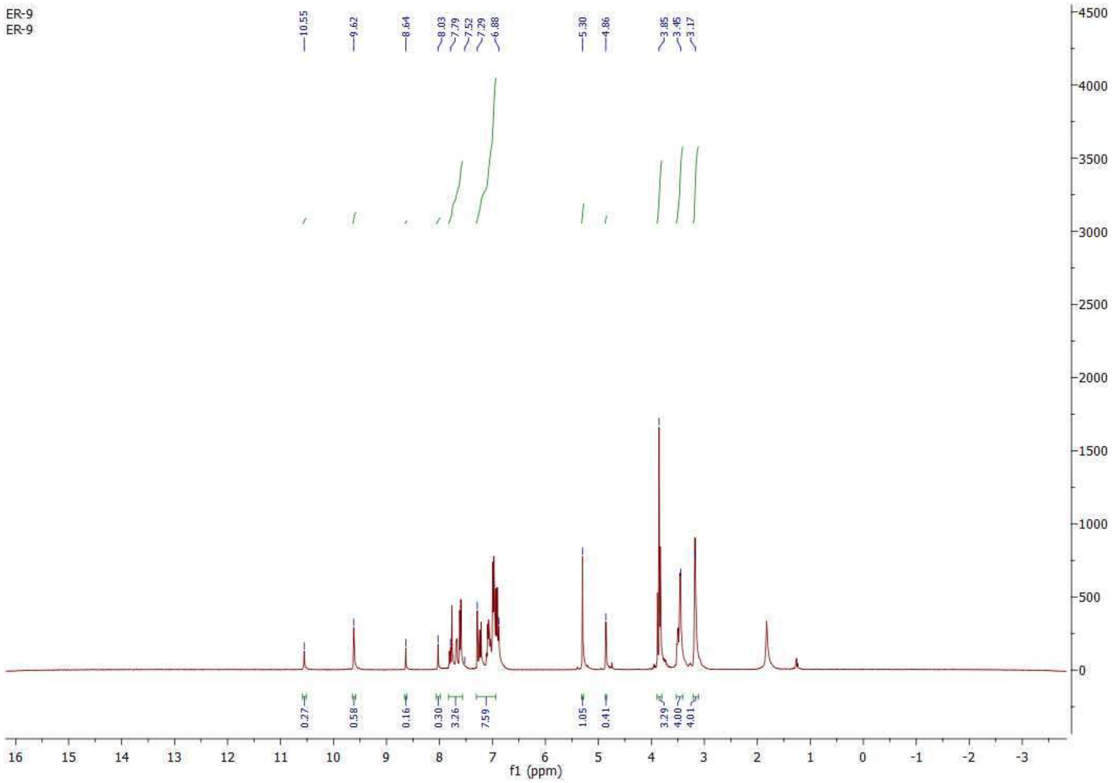

Data 11. ^{13}C-NMR spectrum of compound T11

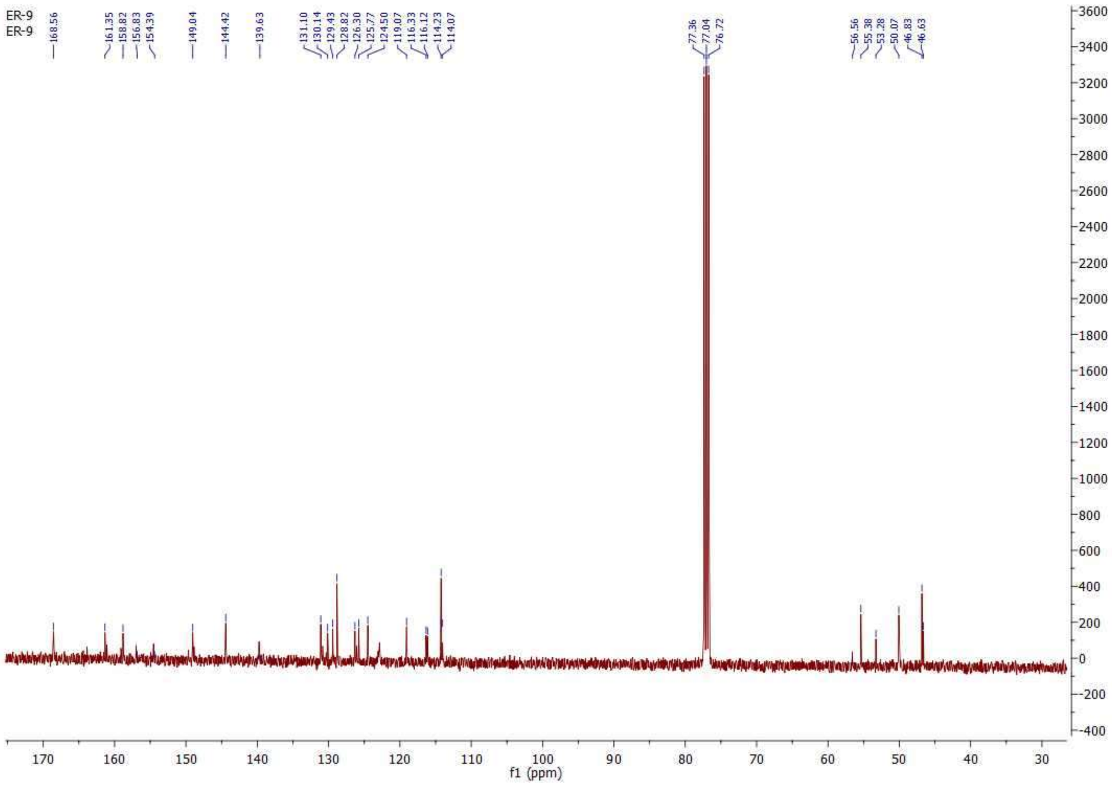

Data 12. HRMS spectrum of compound T11

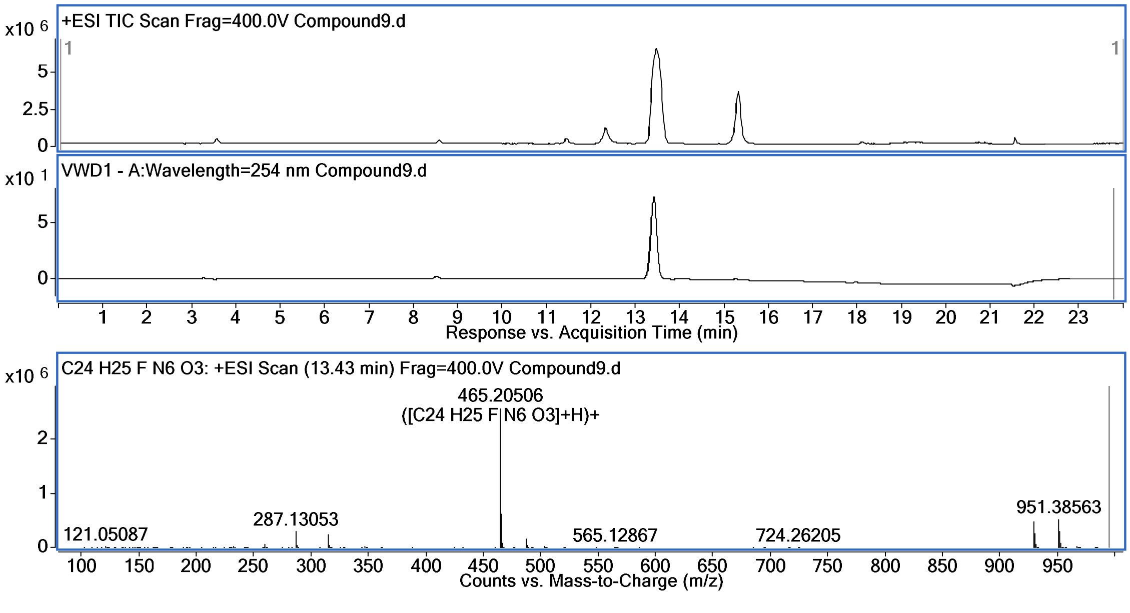

Data 13. ^{1}H-NMR spectrum of compound T12

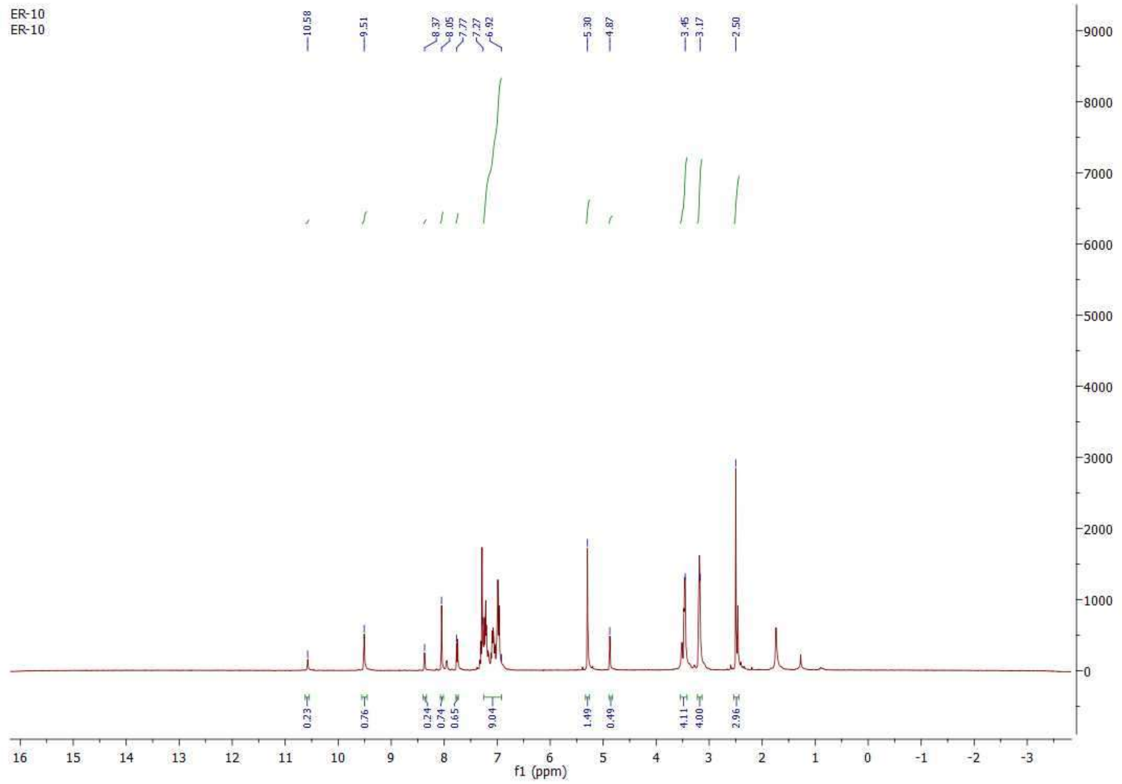

Data 14. ^{13}C-NMR spectrum of compound T12

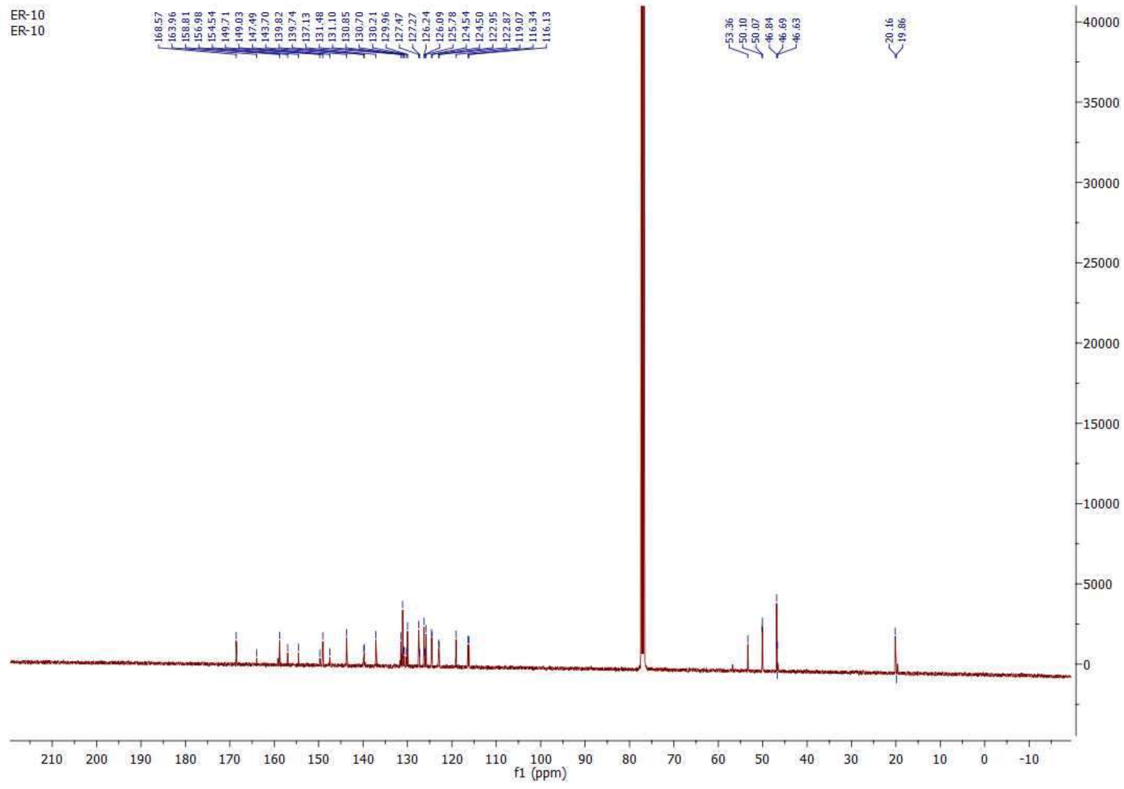

Data 15. HRMS spectrum of compound T12

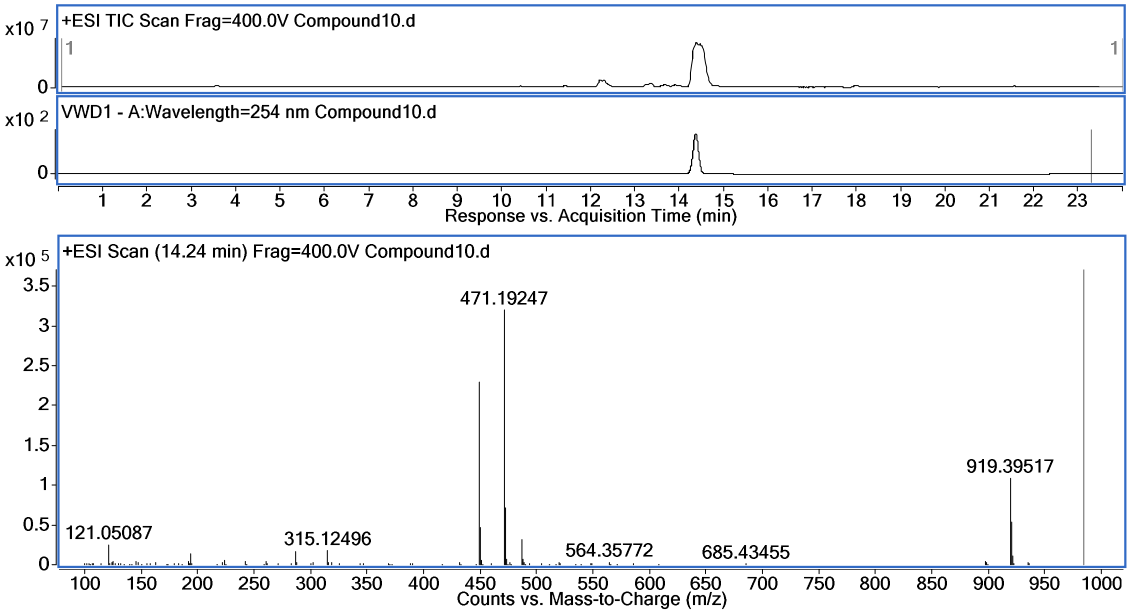

Supplement: Supplementary file 1 [file molecules-25-05371-s001.pdf]
